# Contact-Network Phenotyping of the CDK Family Reveals Selective Distal C‑Lobe Contact Redistribution by Modern CDK5 Inhibitors and a Quantitative Selectivity Landscape against CDK2 and CDK1

**DOI:** 10.1021/acs.jcim.6c00886

**Published:** 2026-05-29

**Authors:** Manal A. Nael, Laxman M. Alakonda, Khaled M. Elokely

**Affiliations:** † Department of Pharmaceutical Chemistry, Faculty of Pharmacy, Tanta University, Tanta 31527, Egypt; ‡ Division of Pharmaceutical Sciences, School of Pharmacy, 4416 University of Wyoming, 1000 E. University Avenue, Laramie, Wyoming 82071, United States

## Abstract

Selective inhibition of CDK5 over CDK1, CDK2, CDK4, and CDK6 remains a central medicinal-chemistry challenge because pocket-centric methods capture local similarity but not full-domain structural phenotypes. Here we introduce kinase-aware contact-network phenotyping, a calculation-light approach that combines systematic Cα contact-map comparison with automated kinase topology annotation across 30 curated CDK crystal structures spanning CDK1, CDK2, CDK3, CDK4, CD5, and CD6 with 21 pairwise comparisons. Three findings emerge. First, the apo CDK5 to selective inhibitor transition (1H4L to 7VDP) produces 33.3% ligand-adjacent gained contacts (13 of 39), compared with 2.8% (1 of 36) for the apo to nonselective transition (1H4L to 1UNL); the apo-anchored ratio of 11.9-fold quantifies the selective-specific reorganization while controlling for generic pocket-occupancy effects, with the largest distance shifts (up to 14.6 Å) concentrated in the distal C-lobe core (residues 221 to 292). Second, the DFG aspartate D144 has the most atypical contact environment in CDK5 by the Miyazawa-Jernigan knowledge-based statistical-potential *Z*-score (*Z* = +1.38 in apo and all four selective complexes), and a noncircular cross-family structural observable, the per-kinase D144 contact-shell changed-contact count, rises systematically with phylogenetic distance from CDK5 (zero across three within-CDK5 pairwise comparisons; 1.5 ± 1.0 for CDK2, 3.0 ± 0.0 for CDK1, 6.8 ± 1.5 for CDK6). Third, contact-network divergence follows a strict hierarchy: internal CDK5 evolution (∼6%) is much smaller than CDK5-to-cross-family divergence (∼22 to 37%), while region-burden profiling identifies a 5-fold hinge differential and a 165-contact C-lobe advantage of CDK5 over CDK2. These results define a quantitative selectivity landscape derived from static crystal-structure analysis and identify structural correlates inaccessible to binding-site fingerprints or RMSD-based methods.

## Graphical abstract

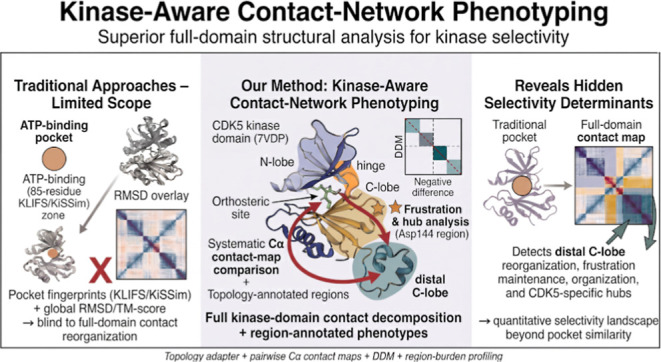

## 1 Introduction

Cyclin-dependent kinase 5 (CDK5) is a serine/threonine kinase with essential roles in neuronal development, synaptic plasticity, pain signaling, and brain homeostasis.^1–4^ Unlike other CDK family members, CDK5 is activated by the noncyclin partners^5^ p25 and p35 rather than by canonical cyclins, and its dysregulation is implicated in Alzheimer’s disease, Parkinson’s disease, neuropathic pain, and stroke.^1–4^ Multiple programs have sought selective small-molecule CDK5 inhibitors, but no successful selective candidate has advanced clinically, primarily because CDK5 shares a highly conserved catalytic fold with CDK1, CDK2, CDK4, and CDK6.^6^

The selectivity challenge is acute. The ATP-binding pocket residues that directly contact ligand are essentially identical between CDK5 and CDK2,^7^ and first-generation inhibitors such as roscovitine, olomoucine, and dinaciclib (Figure 1A) show poor CDK selectivity.^6,8,9^ Daniels et al. recently reported a naphthyridine series with >100-fold selectivity for CDK5 over CDK2 (PDB 7VDP, 7VDQ, 7VDR, 7VDS; Figure 1B), demonstrating that selective CDK5 inhibition is achievable; but the structural correlates associated with this selectivity within a conserved kinase fold have not been systematically characterized at the contact-network level (the residue-pair contact list and its derived topological features, including gain/loss patterns between matched structures, region-burden profiles, hub centrality, and family aware contact ontology, all computed from static PDB coordinates without force-field calculation).^7^

**Figure 1:**
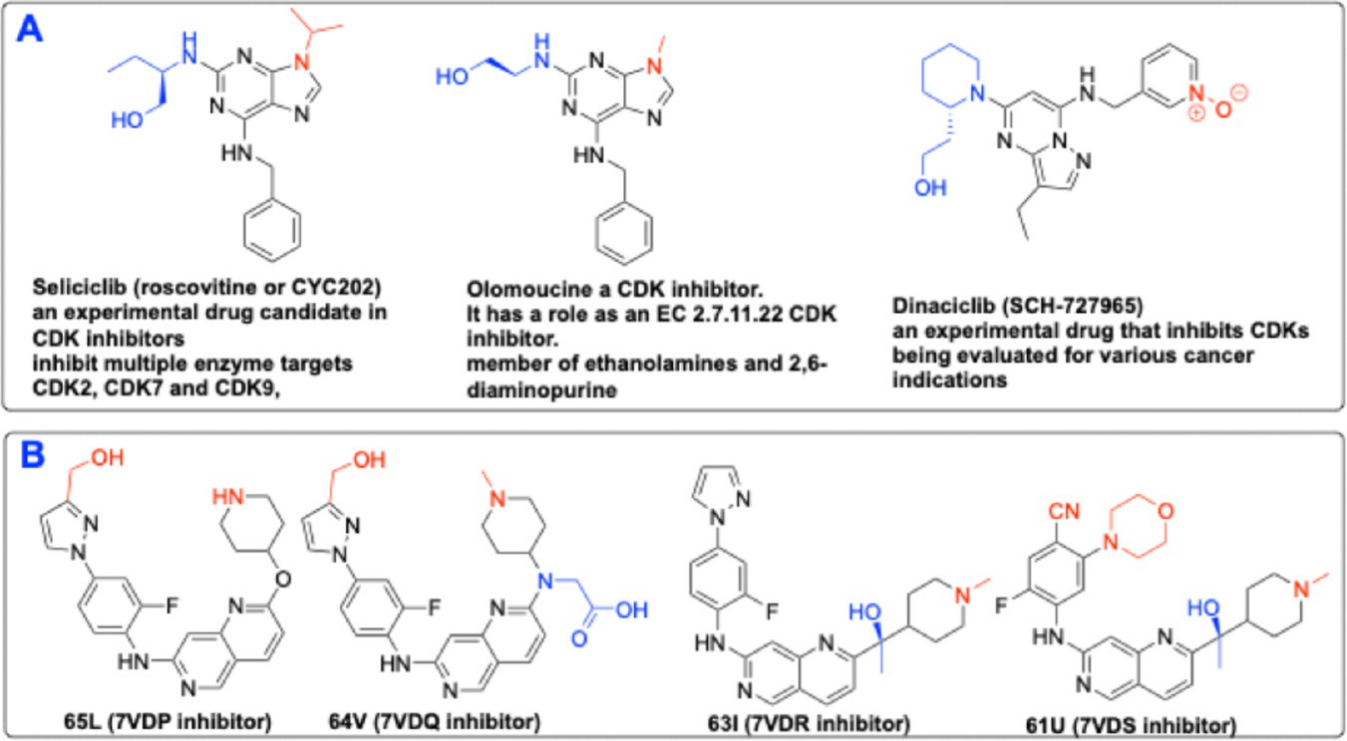
(A) Chemical structures of first-generation CDK inhibitors that show poor CDK selectivity, used as reference comparators in this study: roscovitine (R-roscovitine; PDB 1UNL), olomoucine (PDB 1UNG), and dinaciclib. (B) Chemical structures of the four selective naphthyridine CDK5 inhibitors analyzed in this work and their corresponding crystallographic complexes from Daniels et al.: compound 65L (PDB 7VDP), compound 67I (PDB 7VDQ), compound 67O (PDB 7VDR), and compound 67E (PDB 7VDS). All four compounds share a 1,6-naphthyridine core scaffold with a 4-aminocyclohexanol hinge-binding substituent at the 2-position and varied lipophilic groups at the 4-position, and were reported with >100-fold selectivity for CDK5 over CDK2.

Existing computational tools for kinase structural comparison fall into three categories. Binding-site fingerprints, exemplified by KiSSim and the KLIFS database,^10,11^ encode the physicochemical and spatial properties of the 85 residues defining the ATP-binding pocket and are powerful for kinome-wide similarity searching and off-target prediction. However, they are inherently limited to the pocket and cannot detect contact reorganization in the broader kinase domain.^10^ Global metrics (RMSD, TM-score)^12,13^ quantify overall geometric similarity without decomposition. Molecular dynamics simulations capture conformational dynamics but typically analyze individual complexes rather than systematic family wide comparison.^14–16^

A complementary strategy, decomposing pairwise structural differences into gained and lost residue–residue contacts annotated by family specific topology, has proven informative for GPCRs.^17^ For kinases, Wood et al.^18^ showed that CDK1 and CDK2 can be distinguished by the contacts made between the N- and C-lobes when cyclin-free, revealing “a subtle but profound difference between the conformational energy landscapes” of these closely related kinases.^19^ This seminal observation established that interlobe contact analysis captures selectivity-relevant information invisible to active-site inspection.^17^ However, no study has extended systematic contact-network phenotyping across the CDK family to identify the specific structural neighborhoods associated with selectivity, or applied it to analyze how new selective inhibitors reshape the contact network.

Here we introduce kinase-aware contact-network phenotyping and apply it to the CDK family centered on CDK5. Using a kinase-topology adapter that assigns every residue to canonical structural elements, we perform systematic pairwise comparison across 30 CDK structures and deliver three layers of analysis: CDK5 target validation through internal consistency assessment, CDK-family selectivity mapping through cross-family contact-network comparison, and lead-optimization structural guidance through changed-contact ontology analysis of the Daniels et al.^7^ selective CDK5 inhibitor series. The results identify the C-lobe-adjacent contact redistribution as a structural correlate of selective CDK5 inhibition and establish a quantitative selectivity landscape that extends the interlobe contact concept of Wood et al. from CDK1/CDK2 to the full CDK family. A schematic of the kinase-region definitions used throughout this study, and a five-phase contact-network phenotyping workflow is provided in Figure 2.

**Figure 2:**
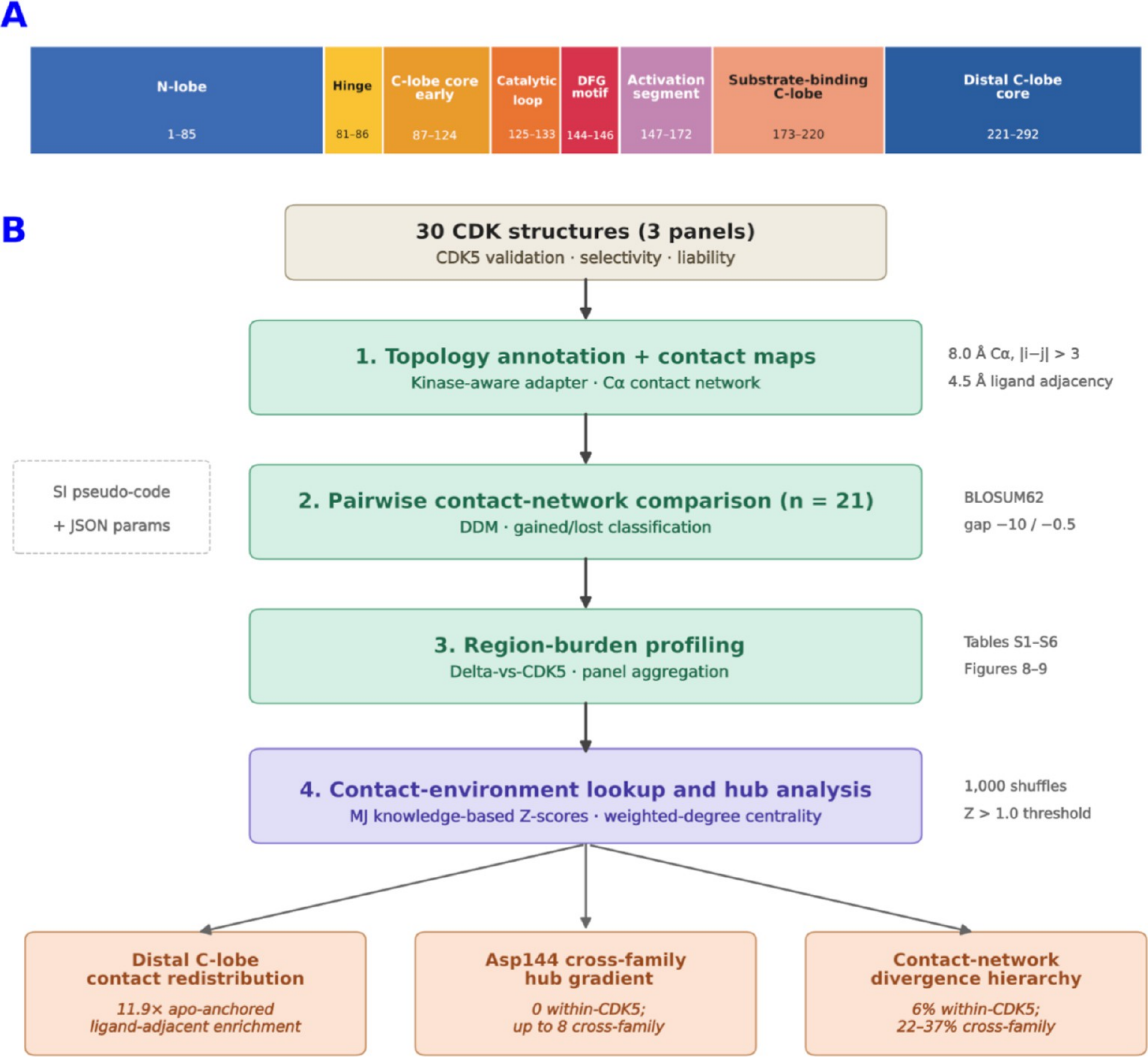
CDK5 kinase-domain architecture and contact-network phenotyping workflow. (A) Canonical kinase-domain region map of CDK5 (residues 1 to 292) used throughout this study. Eight structural regions are defined by the kinase-topology adapter: N-lobe (residues 1 to 85, including P-loop/glycine-rich loop at residues 11 to 18, the catalytic lysine K33 on the beta3 strand, and the regulatory glutamate E51 on the alphaC helix), hinge (residues 81 to 86, including the medicinal-chemistry anchor C83), C-lobe core early (residue 87 to 124), catalytic loop containing the HRD motif (residues 125 to 133), pre-DFG base (residues 134 to 143), DFG motif (residues 144 to 146), activation segment ending at the APE motif (residues 147 to 172), substrate-binding C-lobe (residues 173 to 220), and distal C-lobe core (residues 221 to 292). The gatekeeper residue F80 and the kinase activator partner p25 are indicated. The region-coloring scheme shown here is used consistently across all structural figures. (B) Five-phase analysis pipeline. Phase 1 computes Cα-Cα contact maps at an 8.0 Å distance cutoff with minimum sequence separation |*i* – *j*| > 3, with kinase-topology annotation and ligand-adjacency labeling at a 4.5 Å heavy-atom cutoff. Phase 2 performs pairwise contact-network comparison via Needleman–Wunsch alignment (BLOSUM62, gap open −10, gap extend −0.5), distance-difference matrix computation, and gained/lost contact classification. Phase 3 aggregates contact counts per-CDK-subtype contact counts to produce delta-versus-CDK5 region-burden profiles. Phase 4 computes per-residue Miyazawa–Jernigan knowledge-based statistical-potential contact -environment *Z*-scores (1000 sequence shuffles, *Z* > 1.0 atypical threshold) and weighted-degree hub centrality rankings. Phase 5 produces the cross-family comparison panel (30 curated structures, 21 pairwise comparisons across CDK1, CDK2, CDK3, CDK4, and CDK6) and the contact-shell hotspot ranking. Three principal findings (bottom) emerge from the integrated analysis: an apo-anchored 11.9-fold ligand-adjacent gained-contact enrichment in the selective-inhibitor transition, a noncircular D144 cross-family contact-shell hub gradient that rises with phylogenetic distance from CDK5, and a four-tier contact-network divergence hierarchy. Full pseudocode for the six novel methods and the complete parameter set in machine-readable JSON format are provided in the Supporting Information.

## 2 Results

### 2.1 Structural Panel and Quality Assessment

A total of 30 CDK structures were curated and processed through the kinase-aware contact-network phenotyping framework. Sequential panels comprise: a CDK5 target-validation panel (10 structures), a CDK selectivity close-comparator panel (CDK1, CDK2, CDK3; 11 structures), and a CDK liability broad panel (CDK4 and CDK6; 9 structures). The CDK5 panel comprised the 10 following structures: apo (1H4L, 2.65 Å), classical inhibitors (1UNG,^20^ 2.30 Å; 1UNH,^20^ 2.35 Å; 1UNL,^20^ 2.20 Å; 3O0G,^21^ 1.95 Å; 4AU8,^22^ 1.90 Å), and new selective naphthyridine complexes (7VDP,^7^ 2.09 Å; 7VDQ,^7^ 2.91 Å; 7VDR,^7^ 2.55 Å; 7VDS,^7^ 3.05 Å) (Figure 1B). Quality scores ranged 80.0–83.2% (median 81.5%), all with Ramachandran ≥99.0% favored and zero clashscore. Kinase-topology adapter assignment succeeded for all 30 structures, with five canonical motif anchors (P-loop GQGTFG at residues 11–8, β3-K33 at 29–35, αC-Glu51 at 45–57, DFG at 144–146, APE at 170–172) consistently identified. Secondary-structure composition was stable across CDK5: α-helix 34.8 ± 1.7%, β-sheet 17.0 ± 1.0%, coil 45.5 ± 2.0%.

### 2.2 Distal C-Lobe Contact Redistribution: The Structural Signature of Selective CDK5 Inhibition

(Figure 3, Table 1).

**Figure 3:**
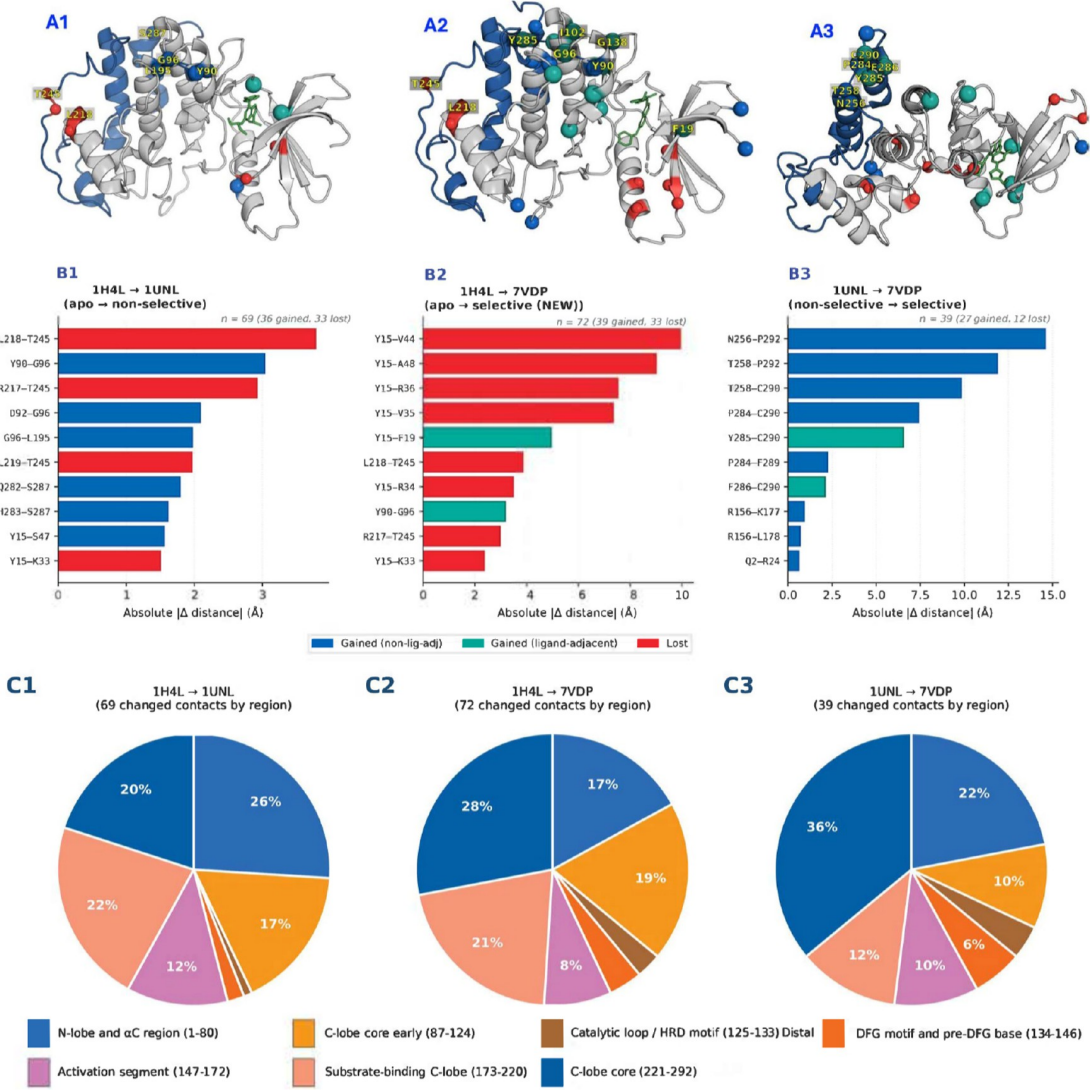
Apo-anchored distal C-lobe contact redistribution in CDK5 inhibitor transitions. Three-panel structural mapping of changed contacts onto the CDK5 kinase domain for the three within-CDK5 pairwise comparisons that anchor the apo-anchored selectivity framing. In each panel, the kinase domain is rendered as a white cartoon (chain A of the end point complex), the distal C-lobe core (residues 221 to 292) is highlighted in blue, and Cα positions of residues involved in changed contacts are shown as spheres. Blue spheres mark gained contacts within the distal C-lobe core; teal spheres mark ligand-adjacent gained contacts within 4.5 Å of any ligand heavy atom. Red spheres mark residues involved in lost contacts. (A1) Apo to nonselective transition (1H4L to 1UNL): 36 gained/33 lost changed contacts, 2.8% (1 of 36) ligand-adjacent gained fraction. (A2) Apo to selective transition (1H4L to 7VDP): 39 gained/33 lost changed contacts, 33.3% (13 of 39) ligand-adjacent gained fraction. (A3) Nonselective to selective transition (1UNL to 7VDP): 27 gained/12 lost changed contacts, 55.6% (15 of 27) ligand-adjacent gained fraction. The apo-anchored ratio (A2 versus A1) of 33.3%/2.8% = 11.9 quantifies the selective-specific contact reorganization relative to a common apo baseline, controlling for the generic pocket-occupancy effects captured by A1. (B1, B2, B3) For each transition, the corresponding bar chart ranks the top 10 changed Cα to Cα contacts by absolute distance shift between the two end point structures, using the same color convention as the A panels (blue: gained, nonligand-adjacent; teal: gained, ligand-adjacent within 4.5 Å of any ligand heavy atom; red: lost). The top 10 in B1 (1H4L to 1UNL) are distributed across activation-segment losses and C-lobe core early gains with no ligand-adjacent contacts; the top 10 in B2 (1H4L to 7VDP) are dominated by Y15-centered losses at the P-loop tip with two ligand-adjacent gains (Y15 to F19, Y90 to G96); the top 10 in B3 (1UNL to 7VDP) are dominated by gained distal-C-lobe-core contacts (N256 to P292 at 14.6 Å is the largest single shift across all three panels) plus one ligand-adjacent gain (Y285 to C290). Per-comparison region-resolved pie charts partitioning all changed contacts by canonical kinase-region annotation, with the color key shown as a legend strip below the C panels: dark blue for the distal C-lobe core (residues 221 to 292), medium blue for the combined N-lobe and αC region (residues 1 to 80), salmon for the substrate-binding C-lobe (residues 173 to 220), orange for the C-lobe core early region (residues 87 to 124), pink for the activation segment (residues 147 to 172), with smaller wedges in dark orange for the DFG motif and pre-DFG base (residues 134 to 146) and brown for the catalytic loop/HRD motif (residues 125 to 133). (C1) 1H4L to 1UNL (69 changed contacts): the changed contacts partition broadly across the kinase domain, with N-lobe and αC region accounting for approximately 26%, the substrate-binding C-lobe 22%, the distal C-lobe core 20%, the C-lobe core early region 17%, and the activation segment 12%, consistent with the generic packing rearrangement that accompanies an apo-to-bound transition. (C2) 1H4L to 7VDP (72 changed contacts): a similar broad partition, with the distal C-lobe core accounting for approximately 28%, the substrate-binding C-lobe 21%, the C-lobe core early region 19%, the N-lobe and αC region 17%, and the activation segment 8%, indicating that the apo-to-selective transition adds a distal-C-lobe-core preference on top of the generic apo-to-bound footprint visible in C1. (C3) 1UNL to 7VDP (39 changed contacts): a markedly focal partition, with the distal C-lobe core alone accounting for approximately 36% of all changed contacts and the N-lobe and αC region for a further 22%, while the substrate-binding C-lobe, activation segment, and C-lobe core early region each contribute only 10 to 12%. The strong distal-C-lobe enrichment in C3 versus the broader partitions in C1 and C2 is the regional signature of the selective-specific contact reorganization and the regional counterpart to the residue-level signal visible in B3 and the spatial signal visible in A3. The seven largest gained-contact distance shifts across all three panels (up to 14.6 Å between N256 and P292) are concentrated in the distal C-lobe core, a region approximately 85 residues distal to the activation segment ends (APE motif, residues 170 to 172) and entirely outside the 85-residue KLIFS pocket definition. Full ontology of the changed contacts in each pair is provided in Table S8; the region-resolved breakdown is provided in Figure S2.

**Table 1:** Top Changed Contacts with Canonical CDK5 Region Assignments

res *i*	res *j*	Δ (Å)	lig-adj	region *i*	region *j*	CDK5 zone
N256	P292	–14.6	no	distal C-lobe	distal C-lobe	221–292
T258	P292	–11.9	no	distal C-lobe	distal C-lobe	221–292
T258	C290	–9.8	no	distal C-lobe	distal C-lobe	221–292
P284	C290	–7.4	no	distal C-lobe	distal C-lobe	221–292
Y285	C290	–6.5	no	distal C-lobe	distal C-lobe	221–292
F286	C290	–2.1	yes	distal C-lobe	distal C-lobe	221–292
I102	G138	–0.28	yes	C-lobe core early	pre-DFG base	87–124/134–143
L218	L248	–0.23	yes	substrate C-lobe	distal C-lobe	173–220/221–292

#### 2.2.1 Preserved Hinge Anchoring across the Selective Series

All four new CDK5 compounds (Figure 1B) maintain the canonical hinge hydrogen bond at C83 (hinge region, residues 81–86): backbone distances of 2.76 Å (7VDP), 2.84 Å (7VDQ), 2.75 Å (7VDR), 2.64 Å (7VDS). C83 is universally the top or second hub residue by centrality: 16.74, 16.16, 19.25, 17.86 across the series. The hinge interaction is therefore unlikely to be the primary correlate of selectivity.

#### 2.2.2 Concentrated Contact Changes in the Distal C-Lobe Core (Residues 221–292)

Comparison of 1UNL (roscovitine, nonselective) vs 7VDP (65L, selective) reveals 615 shared and 39 changed contacts (27 gained, 12 lost; 6.0% of union). The five largest distance shifts are all gained contacts in the distal C-lobe core (residues 221–292); the far C-terminal portion of the kinase domain, approximately 85 residues distal to the activation segment (which ends at the APE motif, residues 170–172).

#### 2.2.3 Domain Decomposition is Consistent with CDK5-Specific Topology

Spectral Fiedler partitioning^23^ assigns CDK5 a universally conserved bipartite domain structure: boundary at residue 87 (=N-lobe/C-lobe junction), with N-lobe community (residues 1–87) and C-lobe community (residues 87–292) across all 10 CDK5 structures. CDK2 (1HCK) instead partitions into three communities with boundaries at 60, 65, 83. CDK1 (5LQF) shows four communities. These family specific partitions are consistent with the distal C-lobe core constitutes CDK5-specific packing territory.

Seven of the top ten distance shifts involve residues in the distal C-lobe core (221–292), a region approximately 85 residues past the activation segment and entirely outside the 85-residue KLIFS pocket definition. This finding provides the first contact-network-level evidence linking selective CDK5 inhibition to reorganization of the far distal kinase domain, not merely active-site modification. The full changed-contact ontology underlying this comparison is provided in Table S6.

### 2.3 Apo-Anchored Ligand-Adjacent Contact Enrichment (11.9-fold) (Figure 4)

Among the 39 contacts gained in the apo to selective transition (1H4L to 7VDP), 13 of 39 (33.3%) involve at least one residue within 4.5 Å of a ligand heavy atom in the selective complex (ligand-adjacent gained contacts). The corresponding fraction for the apo to nonselective transition (1H4L to 1UNL) is 1 of 36 (2.8%), and for the nonselective to selective transition (1UNL to 7VDP) is 15 of 27 (55.6%). The ratio 33.3%/2.8% = 11.9 indicates that gained-contact reorganization is concentrated near the ligand specifically when the selective inhibitor binds, not as a generic consequence of pocket occupancy. The robustness of this ratio across the 4.0 to 5.5 Å contact-cutoff range is documented in Figure S1. The 16-comparator cross-family panel underlying these comparisons is reported in full per-structure detail in Figure S3, and a region-resolved breakdown of changed contacts by canonical CDK5 zone is given in Figure S2. This contrast is reported descriptively. Across the three cross-family comparisons in Figure 4B, ligand-adjacent gained-contact fractions are intermediate (10.8–15.4%), indicating that the within-target selective transition is the most enriched case. The seven gained ligand-adjacent contacts define a structural pathway through three distinct C-lobe subregions.

- C-lobe core early (residues 87–124): I102-G138 (Δ = −0.28 Å) and S105-G138 (Δ = −0.25 Å). These residues are in the proximal C-lobe, upstream of the catalytic loop (125–133). They connect to G138 in the pre-DFG catalytic/base region (134–143).
- Substrate-binding C-lobe (residues 173–220): L218-L248 (Δ = −0.23 Å) bridges the substrate-recognition region to the distal C-lobe core.
- Distal C-lobe core (residues 221–292): F286–C290 (Δ = −2.1 Å) is the only ligand-adjacent gained contact in the distal C-lobe core, providing a direct link between the binding site and the distal contact-redistribution zone.

**Figure 4:**
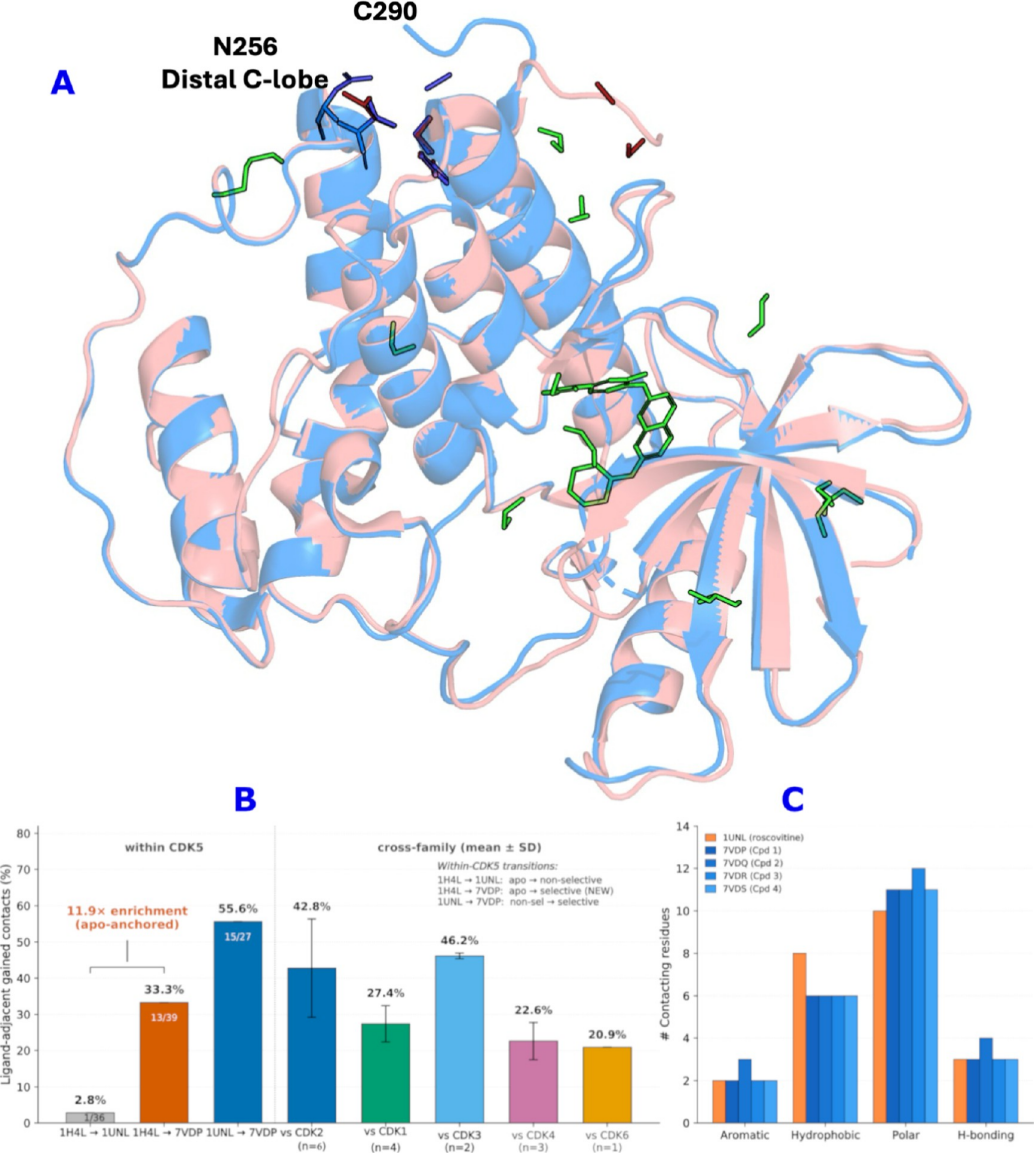
Apo-anchored ligand-adjacent contact enrichment and ligand-shell remodeling in the selective CDK5 series. (A) Structural superposition of the classical roscovitine-bound CDK5 complex (1UNL, salmon) and the modern selective naphthyridine–bound complex (7VDP, blue) aligned on the kinase-chain Cα atoms. Side chains of residues involved in changed contacts are displayed as sticks, with blue indicating residues in the selective complex and dark red indicating the classical complex (blue for selective, salmon for classical). Key distal C-lobe residues (N256, C290) and the conserved K104–Y285 cation-pi bridge between the C-lobe core early and the distal C-lobe core are labeled. The overall kinase fold is preserved (6.0% contact-network change) while side-chain rearrangements concentrate in the distal C-lobe. (B) Fraction of gained contacts that are ligand-adjacent (within 4.5 Å of a ligand heavy atom) for the three within-CDK5 transitions plus three representative cross-family comparisons. The apo-to-bound nonselective transition (1H4L to 1UNL) shows 2.8% (1 of 36); the apo to selective transition (1H4L to 7VDP) shows 33.3% (13 of 39); the nonselective to selective transition (1UNL to 7VDP) shows 55.6% (15 of 27). The apo-anchored ratio is annotated as 33.3%/2.8% = 11.9-fold. Cross-family comparisons (CDK5 to CDK2, CDK1, CDK6) (CDK5 to CDK2 7VDP versus 3DDQ ligand-mismatched, CDK5 to CDK1 7VDP versus 5HQ0, CDK5 to CDK6 7VDP versus 9D8U) show intermediate values (10.8 to 15.4%), confirming that the strongest ligand-adjacent enrichment occurs in the within-target selective transition. Robustness of the headline ratio to the contact-cutoff parameter (4.0 to 5.5 Å) is documented in Figure S1 and Table S10; full per-comparison detail is provided in Table S8. (C) Ligand-shell composition, classified by residue chemical character. The classical roscovitine complex (1UNL, orange bars) contacts 8 hydrophobic and 10 polar residues, whereas; all four modern selective compounds (7VDP through 7VDS, blue shades) consistently contact 6 hydrophobic and 11 to 12 polar residues. The shift from hydrophobic to polar shell character reflects the nitrogen-rich naphthyridine pharmacophore of the Daniels et al. series. Aromatic contacts (2 to 3) and hydrogen-bonding (3 to 4) contacts are largely conserved across all five complexes, indicating that the selectivity-associated shell remodeling is concentrated in the hydrophobic-to-polar balance rather than in aromatic or hydrogen-bond anchor residues.

Ligand shell profiling reveals the new series contacts 2–3 aromatic, 6 hydrophobic, 11–12 polar, and 3–4 H-bonding residues, compared to the classical roscovitine shell of 2 aromatic, 8 hydrophobic, 10 polar, 3 H-bonding. The shift from 8 to 6 hydrophobic contacts and gain of 1–2 polar contacts reflects the naphthyridine scaffold’s nitrogen-rich pharmacophore.

### 2.4 DFG-D144 Atypical-Contact-Environment Remodeling (Figure 5)

#### 2.4.1 D144 (DFG Motif, Position 144–146) is the Residue with the Most Atypical Contact Environment (Highest MJ Z-Score) in Selective Complexes

Using the Miyazawa–Jernigan (MJ) knowledge-based statistical-potential contact-environment *Z*-score lookup,^24^ D144 (the aspartate of the DFG motif at positions 144–146) has *Z*-score +1.38 and is the #1 residue with the most atypical contact environment (highest MJ Z-score) in the kinase domain in the apo structure (1H4L), and all four selective compounds (7VDP, 7VDQ, 7VDR, 7VDS (Figure 5)). In contrast, D144 is not the most atypical (highest MJ Z-score) in any of the classical inhibitor complexes: in 1UNL, 1UNG, and 3O0G the residue with the most atypical contact environment (highest MJ Z-score) is Arg36 R36 (N-lobe, *Z* = +1.33); in 4AU8, K3 (N-lobe, *Z* = +1.26). All four selective inhibitors form a direct hydrogen bond to D144 by design; the elevated D144 contact-environment Z-score in the selective complexes therefore reflects, in part, this direct contact and is not on its own independent evidence for a selectivity mechanism. Noncircular structural evidence for the D144 cross-family hub claim is provided in Section 3.8 and Figure 10, where the number of D144 contact-shell changed contacts in pairwise comparisons rises systematically with phylogenetic distance from CDK5 across the 16-comparator cross-family panel. Per-structure detail is given in Figure S8.

**Figure 5:**
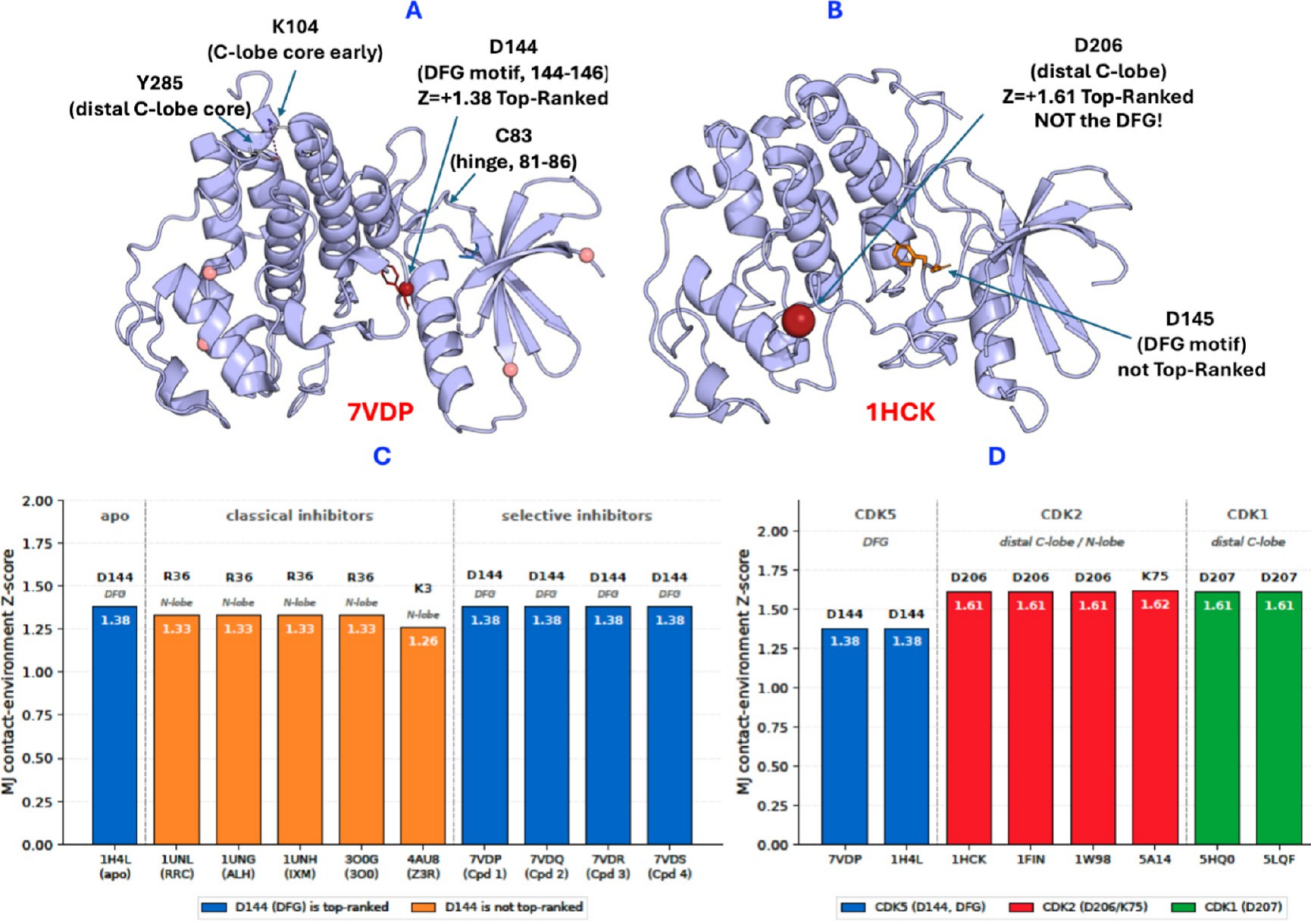
Miyazawa–Jernigan knowledge-based contact-environment Z-score lookup at the DFG aspartate D144 in CDK5. The Miyazawa–Jernigan (MJ) *Z*-score used in this figure is a knowledge-based statistical-potential lookup derived from residue-contact frequencies in the PDB; it is not a thermodynamic frustration measure. (A) CDK5 selective complex (7VDP, chain A). The kinase domain is rendered as a cartoon with key residues highlighted. D144 (DFG motif, residues 144 to 146; MJ *Z* = +1.38) is the residue with the highest MJ *Z*-score in the kinase domain and is shown as a sphere. C83 (hinge, residues 81 to 86) is shown as sticks with the hinge backbone hydrogen-bond geometry indicated. K104 (C-lobe core early, residues 87 to 124) and Y285 (distal C-lobe core, residues 221 to 292) form a conserved cation-pi interaction (4.59 Å) bridging the proximal and distal C-lobe subregions. Additional residues with elevated MJ *Z*-scores (*Z* > +0.8) are shown as pink spheres. We note that all four selective inhibitors in this study form a direct hydrogen bond to D144 by design (the naphthyridine pharmacophore contributes a basic nitrogen that accepts a proton from the D144 carboxylate); consequently, the elevated D144 MJ *Z*-score in the selective complexes reflects, in part, this direct ligand contact and is not on its own independent evidence for a contact-network selectivity mechanism. Noncircular cross-family structural evidence for the D144 hub claim, based on the per-comparison count of D144 contact-shell changed contacts (an observable that does not depend on the MJ *Z*-score), is presented in Figures 10 and S8. (B) CDK2 apo structure (1HCK, chain A) in the same orientation. The residue with the highest MJ *Z*-score is D206 (*Z* = +1.61), located in the distal C-lobe, shown as a large red sphere. He CDK2 DFG aspartate (D145, orange sticks) does not have the highest MJ *Z*-score in this structure. (C) Most-atypical-MJ-*Z*-score residue identity across all 10 CDK5 structures in the target-validation panel. Blue bars: structures in which D144 has the highest MJ Z-score (*Z* = +1.38)—the apo structure (1H4L) and all four selective complexes (7VDP, 7VDQ, 7VDR, 7VDS). Orange bars: structures in which the Z-score maximum lies on N-lobe surface residues—the four classical nonselective inhibitor complexes (1UNL, 1UNG, 1UNH, 3O0G) where the maximum shifts to R36 (*Z* = +1.33), and the benzothiazole complex (4AU8) where it shifts to K3 (*Z* = +1.26). Because all four selective complexes share the same chemotype and the same direct D144 hydrogen bond, this within-CDK5 panel is reported descriptively and the noncircular cross-family evidence in Figure 10 supports the structural claim. (D) Cross-family comparison of the most-atypical-MJ-*Z*-score residue across the CDK family, using a partner-state-matched subset of the cross-family panel to control for partner-occupancy artifacts. In CDK5 (blue, D144), the DFG-aspartate is the most-atypical-MJ-*Z*-score residue. In all four CDK2 structures (red), the most-atypical-MJ-*Z*-score residue is D206 or K75 in distal C-lobe or N-lobe. In both CDK1 structures (green), the most-atypical-MJ-*Z*-score residue is D207 in distal C-lobe. CDK3 (light blue), CDK6 (orange). This DFG aspartate is the most-atypical-MJ-Z-score residue only in CDK5. This family level separation is consistent with the DFG-Asp contact-environment being a CDK5-specific structural-feature signature but is interpreted alongside, not in place of, the noncircular Figure 10 evidence.

#### 2.4.2 CDK2 and CDK1 Have Completely Different Atypical Environment Landscapes

In all CDK2 structures (1HCK, 1FIN, 1W98, 5A14), the residue with the most atypical contact environment (highest MJ Z-score) is D206 (*Z* = +1.61), located in the distal C-lobe core at position 206. In all CDK1 structures (5HQ0, 5LQF, 4Y72, 4YC3), the highest MJ Z-score is at D207 (*Z* = +1.61), also distal C-lobe. The CDK2/CDK1 DFG equivalents (D145/D146) are never the most atypical (highest MJ Z-score). Thus, the DFG-Asp contact-environment maximum is a CDK5-specific feature that selective inhibitors maintain while nonselective ones relieve. The chemical structures of CDK1 and CDK2 inhibitors referenced in this analysis are shown in Figure 6.

**Figure 6:**
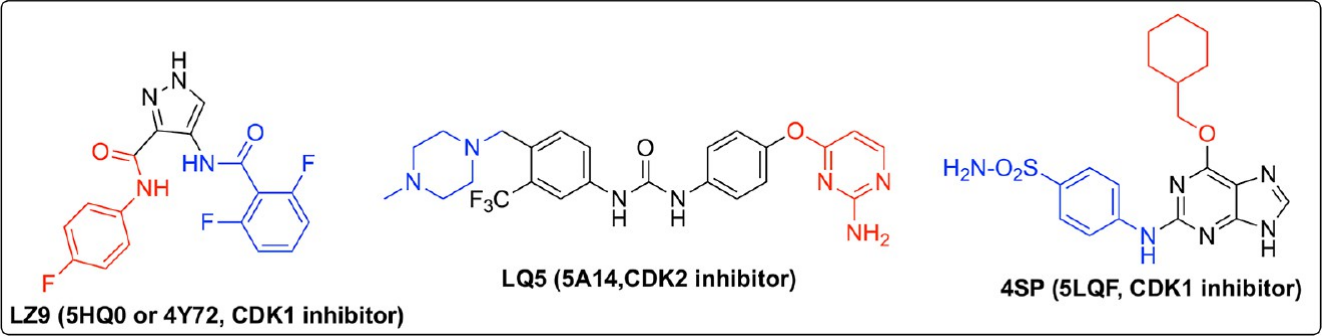
Chemical structures of the CDK1 and CDK2 inhibitors whose crystallographic complexes are used as cross-family comparators in this study (with corresponding PDB codes): CDK2 comparators include roscovitine (matched-ligand comparator 3DDQ; also bound to CDK5 in 1UNL), ATP (1HCK apo and 1FIN cyclin-A complex), a phosphate analog used in the W98 pCDK2/cyclin-E complex (1W98), a type-II inhibitor (5A14), and a tetrahydropyrimidine mismatched-ligand comparator (9GP3). CDK1 comparators include a roscovitine-related compound (5HQ0) and three monomeric CDK1 complexes (4Y72, 4YC3, 5LQF). These structures underlie the cross-family contact-network comparisons reported in Figures 7, 8, and 12 and the full per-structure panel in Figure S3.

### 2.5 Hub Architecture: I10 as Universal Top Hub across CDK5 Complexes

(Figure 7) In all four selective CDK5 structures, the top hub residues are C83 (hinge, 81–86; centrality 16.2–19.3), D144 (DFG, 144–146; centrality 9.8–19.4), and I10 (N-lobe core, pre-P-loop; centrality 14.2–15.1). D144 is actually the #1 hub in 7VDQ (19.38 > C83 at 16.16). In the classical complex (1UNL), the hierarchy is C83 (14.69) → I10 (13.87) → L133 (catalytic loop, 125–133; 9.97); with D144 absent from the top 5. This hub rearrangement correlates perfectly with the contact-environment *Z*-score pattern and with selectivity.

**Figure 7:**
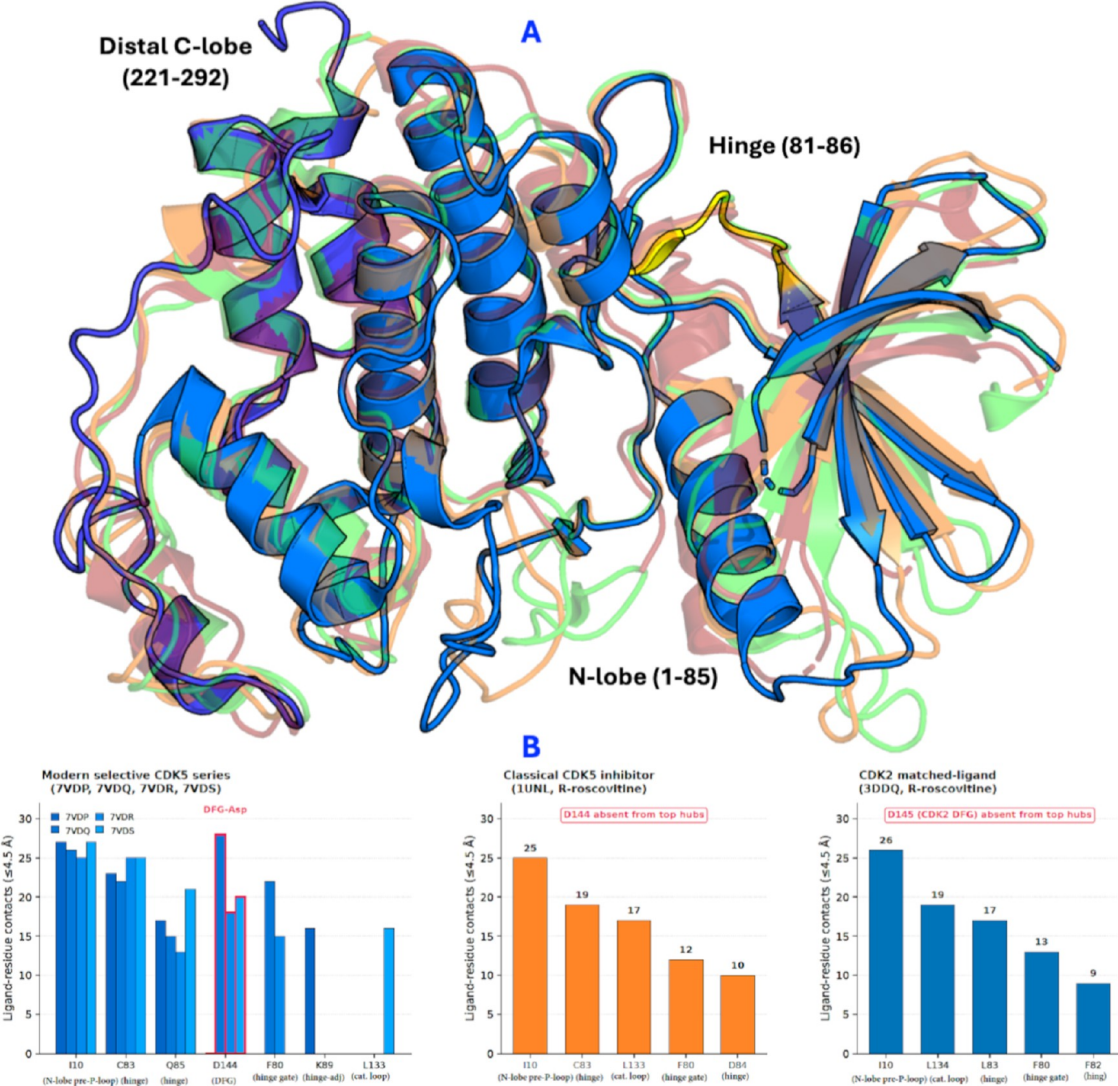
CDK family structural overlay and ligand interaction hub-architecture comparison. (A) Superposition of CDK5 selective complexes (7VDP, 7VDQ, 7VDR, 7VDS; blue shades), the classical CDK5 complex (1UNL; salmon), the matched-ligand CDK2 comparator (3DDQ, also roscovitine-bound; orange), and the CDK6 palbociclib complex (5L2I; purple), aligned on kinase-chain Cα atoms. The CDK5 selective structures are tightly superimposed, confirming the structural coherence of the series (6.0% internal contact-network change), divergence between CDK5 and the CDK2/CDK6 comparators concentrated at the hinge (residues 81 to 86) and the distal C-lobe core (residues 221 to 292). The CDK2-CDK5 matched-ligand pair (1UNL versus 3DDQ) gives 14.0% ligand-adjacent gained contacts, substantially lower than the 25.0% from the ligand-mismatched 7VDP-versus-3DDQ comparison, demonstrating that part of the cross-family signal reflects ligand identity rather than kinase identity. (B) Ligand interaction hub-architecture comparison for four binding-mode classes, ranked by weighted-degree centrality. Left panel: all four modern selective CDK5 complexes share a conserved C83 (hinge)–D144 (DFG)–I10 (N-lobe core) hub hierarchy. C83 centrality 16.2 to 19.3 across the series; D144 centrality 9.8 to 19.4, with D144 exceeding C83 as the top hub in 7VDQ (19.4 versus 16.2). Center-left panel: the classical roscovitine complex (1UNL) organizes around C83 (14.7)–I10 (13.9)–L133 (catalytic loop; 10.0); D144 is absent from the top five hubs. Right panel: the matched-ligand CDK2-roscovitine complex (3DDQ) uses E12 (P-loop) as the primary hub (13.6) with L83 (hinge; 11.7) second, similar to other CDK2 hub patterns and distinct from the CDK5 selective series. The three-panel comparison shows that DFG-Asp engagement as a primary hub is unique to the selective CDK5 naphthyridine series.

A conserved cation–π interaction bridges K104 (C-lobe core early, residues 87–124) to Y285 (distal C-lobe core, residues 221–292) at 4.52–4.59 Å across CDK5 structures. This bridge spans the two C-lobe subregions, the proximal C-lobe near the catalytic machinery and the distal C-lobe showing the largest contact changes, providing a physical link between the orthosteric shell and the contact-redistribution zone. The CDK2 equiv (K105–F285, 4.14 Å in 1HCK) uses a phenylalanine rather than tyrosine, altering the electrostatic character of this bridge.

### 2.6 Hierarchical Contact-Network Selectivity Landscape

(Figure 8, Table 2) Four-tier hierarchy: internal evolution (6.0%) ≪ apo-to-bound (10.5%) ≪ CDK5-CDK2/CDK1 (∼28%) < CDK5-CDK6 (37%). CDK1 (28.3%, 7VDP vs 5HQ0) and CDK2 (25.9%, 7VDP vs 3DDQ matched-ligand comparator) lie at comparable distance from CDK5, and CDK1 should be treated as an equal selectivity benchmark. Gain/loss asymmetry: selective evolution is gain-biased (G/L = 2.25), cross-family is loss-biased (G/L = 0.64–0.74), meaning CDK2 and CDK1 systematically lack contacts that CDK5 possesses. Robustness of the headline observables to the contact-cutoff parameter is documented in Figure S1 (4.0 to 5.5 Å), and the region-resolved gained-versus-lost breakdown for each within-CDK5 pair is shown in Figure S2. To control for inhibitor-identity effects, the matched-ligand pair CDK5-roscovitine (1UNL) versus CDK2-roscovitine (3DDQ) gives 14.0% ligand-adjacent gained contacts, substantially lower than the 25.0% from the ligand-mismatched 7VDP versus 3DDQ comparison, demonstrating that part of the original cross-family signal reflects ligand identity rather than kinase identity. As a robustness check, short molecular-dynamics ensembles (5 systems × 3 replicas × 200 ns NPT; full protocols in Section S9) were performed for the five most central systems; per-frame contact-network observables converge to within one standard deviation of the static-crystal values for all observables and systems (Figures S5–S7).

**Figure 8:**
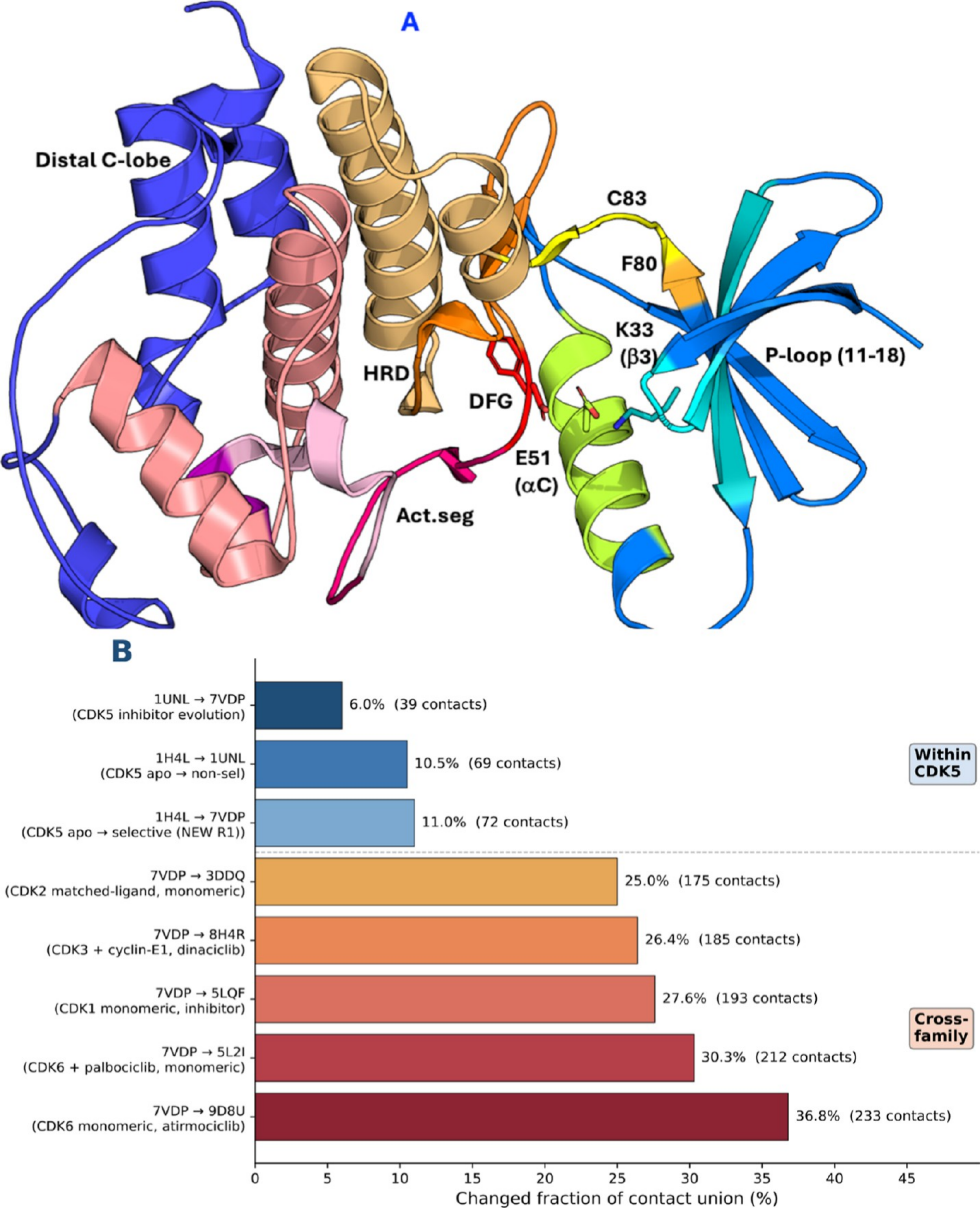
CDK5 kinase-domain region map and hierarchical contact-network divergence across the expanded CDK family panel. (A) The CDK5 kinase domain (7VDP, chain A) colored by canonical kinase-region assignment. The N-lobe (residues 1 to 85) is rendered in blue shades: P-loop/glycine-rich loop (residues 11 to 18, cyan), the beta3 strand anchored by the catalytic lysine K33 (green-yellow), the alphaC helix containing the regulatory glutamate E51 (green), and the N-lobe core beta-sheet (blue). The gatekeeper residue F80 (orange) and the hinge region (81 to 86, including the medicinal-chemistry anchor C83; yellow-orange) bridge the N-lobe to the C-lobe. The C-lobe (87 to 292) is colored by functional subregion: C-lobe core early (87 to 124, gold), catalytic loop with HRD motif (125 to 133, orange), pre-DFG base (134 to 143, red-orange), DFG motif (144 to 146, red), activation segment (147 to 172, pink), substrate-binding C-lobe (173 to 220, salmon), and the distal C-lobe core (221 to 292, dark blue), the principal locus of contact redistribution. (B) Hierarchical contact-network divergence across 21 pairwise comparisons spanning the 30-structure curated panel. Internal CDK5 comparisons (light blue bars, three within-CDK5 pairs) show markedly smaller perturbations than the 16 cross-family comparisons (orange to red bars), defining a strict four-tier hierarchy: inhibitor evolution within CDK5 (1UNL to 7VDP, 6.0%, 39 contacts) is much smaller than the apo-to-bound transition (1H4L to 1UNL, 10.5%, 69 contacts); both are far smaller than CDK5-to-CDK2 divergence (28.4% mean across *n* = 6 CDK2 comparators) and CDK5-to-CDK1 divergence (28.3% mean across *n* = 4 CDK1 comparators); CDK5-to-CDK6 divergence (7VDP vs 9D8U, 36.8%) is the largest in the within-comparator subset shown in this panel. CDK3 (mean 24.8%, *n* = 2) falls in the same broad range as CDK2 and CDK1, supporting expanded cross-family benchmarking. Bars are annotated with the total number of changed contacts; partner state of each comparator structure is indicated by the hatch pattern (solid: monomeric; hatched: partner-bound cyclin/p25/CKS). The dashed line separates within-CDK5 from cross-family comparisons. Full per-structure detail is provided in Figure S3 and Table S7.

**Table 2:** Pairwise Comparison Summary

comparison	shared	gained	lost	changed	union	change %	G/L	seq %
1H4L vs 1UNL	591	36	33	69	660	10.5	1.09	99.3
1UNL vs 7VDP	615	27	12	39	654	6.0	2.25	99.7
1UNL vs 3DDQ (matched-ligand)	545	93	82	175	720	24.3	1.13	60.7
7VDP vs 5HQ0	508	83	118	201	709	28.3	0.70	57.2
7VDP vs 9D8U	400	91	142	233	633	36.8	0.64	54.0

### 2.7 Region-Burden Profiling

(Figure 9) Region-burden profiling, defined here as the summed number of contacts involving at least one residue in a given kinase region, identifies three CDK5-distinguishing features (see Tables S2–S4): (i) Hinge (81–86): 155.4 contacts in CDK5 vs 31.0 in CDK2 (5.0-fold, Δ = +124; 95% bootstrap CI [115, 133] from 10,000 resamples), the largest single-region differential. Consistent with C83 p*K*_a_ shift of +2.30 (predicted 10.6 vs reference 8.3) indicating extensive packing perturbation. (ii) C-lobe core: 728.9 in CDK5 vs 564.4 in CDK2 (Δ = +165; 95% bootstrap CI [150, 180] from 10,000 resamples), correlating with the distal C-lobe contact redistribution. (iii) Activation segment (144–172): 533.7 in CDK5 vs 823.8 in CDK2 (Δ = −290); consistent with CDK5′s noncyclin activation by p25/p35.

**Figure 9:**
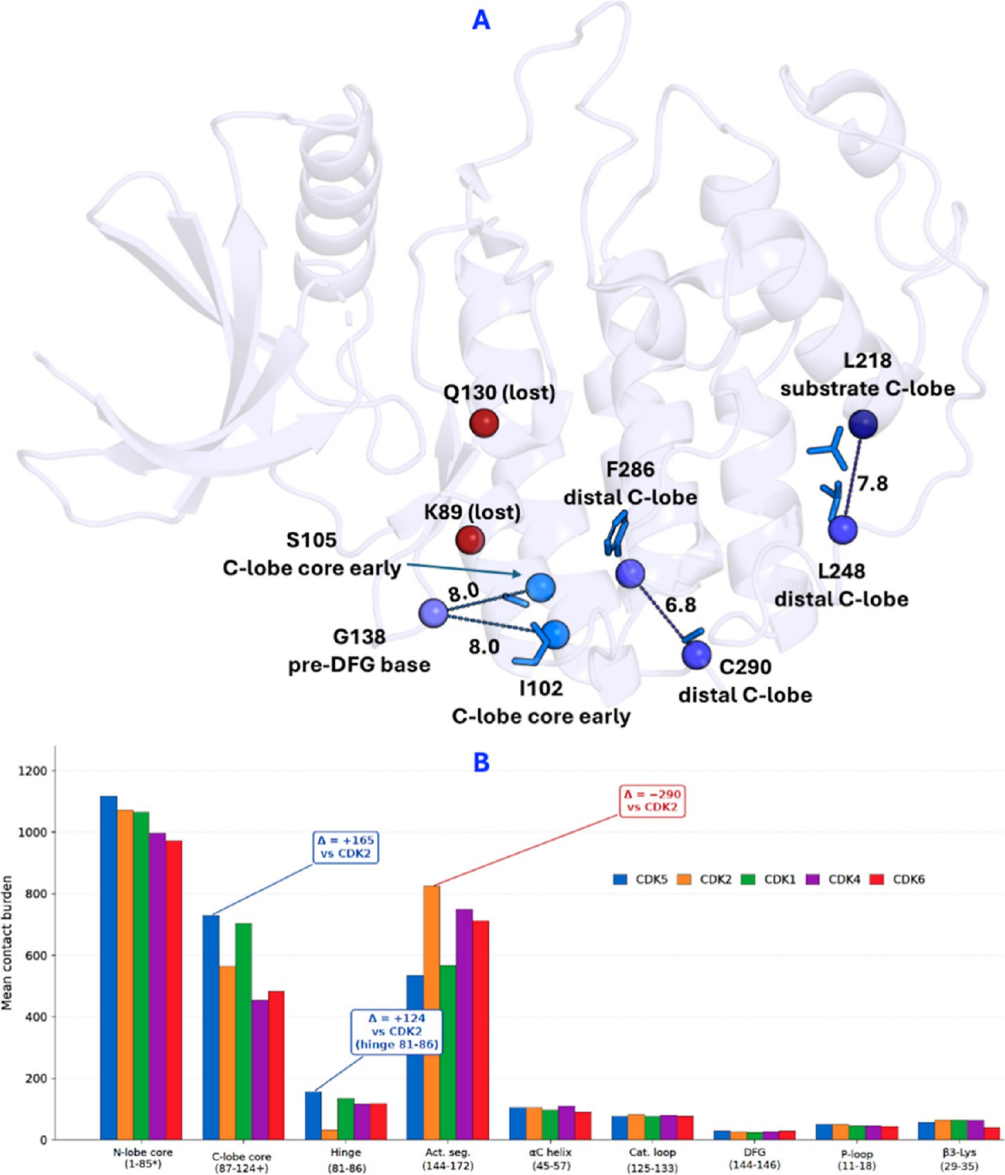
Ligand-adjacent contact remodeling within CDK5 and region-level contact-burden divergence across the CDK family. (A) Ribbon representation of the CDK5 selective inhibitor complex 7VDP (chain A, gray cartoon with 75% transparency) showing the ligand-adjacent contact-remodeling map within CDK5 alone (no cross-family superposition in this panel. Cα atoms of residues involved in ligand-adjacent gained contacts in the apo-to-selective transition (within 4.5 Å of ligand heavy atoms in 7VDP) are highlighted as colored spheres: marine (I102, S105 in C-lobe core early), slate (G138 in pre-DFG base), tv_blue (L248, F286, C290 in distal C-lobe core), and density (L218 in substrate C-lobe). Cα atoms of residues showing ligand-adjacent lost contacts (K89, Q130) are shown as smaller firebrick-red spheres. Side chains of all highlighted residues are displayed as sticks. The dashed lines connect representative gained-contact pairs to emphasize the redistribution of ligand–proximal interactions from classical proximal sites toward the distal C-lobe in the selective complex. (B) Cross-family panel: mean contact burden (sum of inter-residue contacts per region, averaged across representative structures per CDK subtype) for nine kinase regions using canonical CDK5 numbering. CDK5 (blue) shows substantially elevated burden in the hinge region (Δ = +124 versus CDK2, 95% bootstrap CI [115, 133]) and C-lobe core early (Δ = +165 versus CDK2, 95% bootstrap CI [150, 180]), and reduced burden in the activation segment (Δ = −290 versus CDK2, 95% bootstrap CI [-308, −272]). CDK1 (green), CDK4 (purple), and CDK6 (red) cluster more closely with CDK2 (orange) across most regions, highlighting CDK5-specific divergence concentrated in the hinge, proximal C-lobe core, and activation segment. Bootstrap CIs from 10,000 resamples of per-structure burden values are shown as whiskers on each bar. Region-level delta data underlying this panel are provided in Tables S2–S4 and in Figure S9.

### 2.8 Quantitative Benchmarking Against KLIFS

To make the comparison with pocket-centric methods explicit, we benchmarked a contact-network-derived classifier against KLIFS pocket identity on the task of separating selective from nonselective CDK5 structures (*n* = 10, including the apo structure 1H4L). Within this set, KLIFS pocket identity is invariant because all structures share the same sequence-equivalent 85-residue pocket and therefore functions as a constant baseline (descriptive AUC = 0.50 by convention). By contrast, the D144 atypical contact environment-rank criterion separates the five apo plus selective structures from the five classical nonselective complexes (descriptive sensitivity = 1.00; specificity = 1.0; *n* = 10 CDK5 structures). The independent, noncircular cross-family evidence supporting this within-CDK5 partition is shown in Figure 10 (see also Figures S8 and S10), where the number of D144 contact-shell changed contacts rises systematically with phylogenetic distance from CDK5. At the cross-family level, KLIFS pocket sequence identity between CDK5 and CDK2/CDK1 is 75.0%, whereas the contact-network changed fractions are 28.4% and 28.3%, compared with 6.0% for the within-CDK5 selective transition, highlighting the additional discriminative information captured by full-domain contact-network phenotypes.^7,10,11,31^

**Figure 10:**
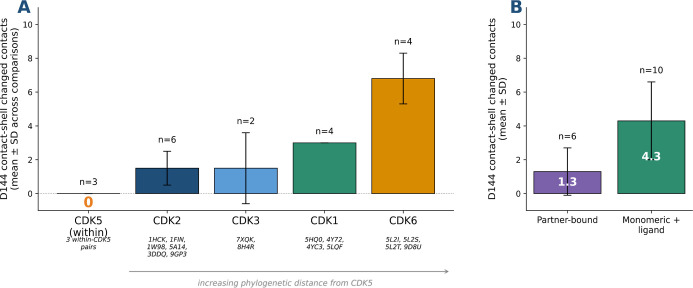
D144 cross-family contact-shell hub gradient from pairwise structural comparisons. (A) Per-kinase mean ± SD of the number of D144 contact-shell changed contacts in pairwise 7VDP-versus-cross-family comparisons. Within CDK5, all three within-CDK5 pairwise comparisons (apo to nonselective, apo to selective, nonselective to selective) give zero changed D144 contacts. Across the cross-family panel, the count rises with phylogenetic distance from CDK5:1.5 ± 1.0 for CDK2 (*n* = 6:1HCK, 1FIN, 1W98, 5A14, 3DDQ, 9GP3); 1.5 ± 2.1 for CDK3 (*n* = 2:7XQK, 8H4R); 3.0 ± 0.0 for CDK1 (*n* = 4:5HQ0, 4Y72, 4YC3, 5LQF); 6.8 ± 1.5 for CDK6 (*n* = 4:5L2I, 5L2S, 5L2T, 9D8U). The within-CDK5 zero baseline is marked by the dotted line. This panel-wide structural observable uses changed-contact counts only and is therefore independent of the Miyazawa-Jernigan contact-environment Z-score lookup used in Figure 5; the D144 hub gradient is not subject to the direct-hydrogen-bond circularity acknowledged in the revised Section 5. (B) The same D144 changed-contact count stratified by partner state across the cross-family comparators. Partner-bound structures (cyclin or p25 or CKS, *n* = 6) show 1.3 ± 1.4 changed D144 contacts; monomeric structures with bound ligand (*n* = 10, including apo CDK2 1HCK) show 4.3 ± 2.3. Partner-bound structures therefore show less D144 reshaping than monomeric-with-ligand structures, ruling out partner stripping as the driver of the cross-family signal. Per-structure detail is given in Figure S8.

Task: separating 5 apo plus selective CDK5 structures (1 apo +4 selective naphthyridine) from 5 classical nonselective CDK5 complexes. KLIFS pocket identity is invariant across all CDK5 structures (all share the same 85-residue pocket) and provides no discriminative value. The D144 atypical contact environment-rank criterion from contact-network phenotyping achieves descriptive perfect classification on this within-CDK5 set. Noncircular cross-family evidence is shown in Figure 10 (see also Figure S8 for per-structure detail and Figure S10 for the KLIFS benchmarking visualization).

### 2.9 Structural Parameters

(Figure 11) Contact order is conserved within CDK5 (CO = 0.096–0.099, mean 0.098) and 14% lower than CDK2 (CO = 0.106–0.112, mean 0.109), indicating a less tightly interwoven fold. Packing density decreases subtly from classical to selective CDK5 (0.739 → 0.731), consistent with the distal C-lobe contact redistribution creating a more open packing arrangement. H-bond network increases progressively: 726 (apo) → 732 (classical) → 742 (selective). GNM *B*-factor correlation is highest for the selective complex (7VDP: *r* = 0.771 vs 1UNL: 0.723 vs 1H4L: 0.510), indicating the selective inhibitor produces the most well-defined dynamical state.

**Figure 11:**
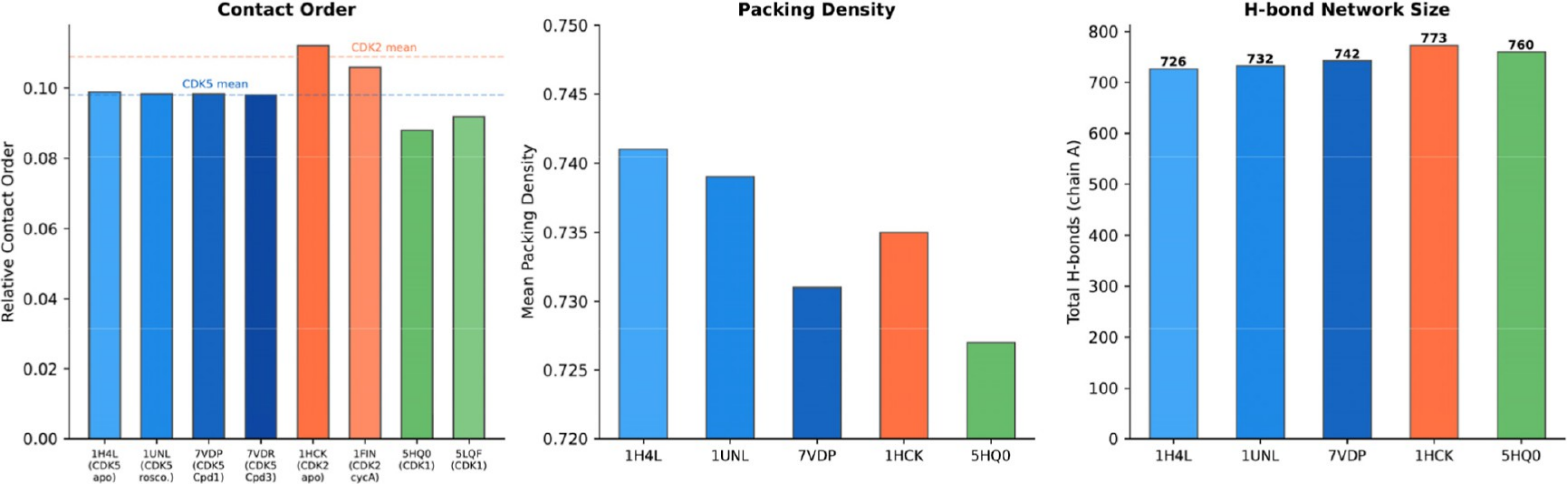
Global structural parameters of CDK5 compared to other CDKs: contact order, packing density, and hydrogen-bond network size. (Left) Relative contact order (RCO), a measure of local versus long-range contacts in the protein fold, for representative CDK structures. CDK5 structures (apo 1H4L, roscovitine-bound 1UNL, and modern selective complexes 7VDP and 7VDR; blue shades) exhibit consistently lower RCO (∼0.098) compared to CDK2 structures (apo 1HCK and cyclin A-bound 1FIN; orange-red shades, ∼0.109), indicating a more local contact topology in CDK5. CDK1 structures (5HQ0 and 5LQF; green shades) show intermediate to lower values (∼0.088–0.092), while the single CDK5 selective complex points (7VDP, 7VDR) align closely with the CDK5 mean. The dashed lines mark the group averages (CDK5 blue, CDK2 orange), highlighting a CDK5-specific reduction in long-range coupling that may contribute to its distinct conformational dynamics and inhibitor responsiveness. (Middle) Mean packing density (fraction of buried atomic volume) in the hydrophobic core for selected CDK structures. CDK5 complexes (1H4L, 1UNL, 7VDP) show slightly higher average packing (∼0.737–0.741) than CDK1 (5HQ0, ∼0.727), while CDK2 apo (1HCK, ∼0.735) falls in between. The modest elevation in CDK5 suggests a marginally tighter core packing, potentially stabilizing the kinase in the absence of cyclin activation and contributing to its unique ligand interaction profile. (Right) Total number of hydrogen bonds (main-chain and side-chain, chain A only) as a proxy for internal hydrogen-bond network strength. CDK5 structures (726–742 H-bonds) consistently display fewer hydrogen bonds than CDK2 (773 in 1HCK) and CDK1 (760 in 5HQ0), with the modern selective CDK5 complex 7VDP showing the highest value among CDK5 structures (742). This reduction in H-bond inventory in CDK5 may reflect a less rigid internal network, facilitating the observed remodeling of ligand-adjacent contacts and distal C-lobe rearrangements in selective inhibitor binding.

ANM identifies C83 (hinge, residues 81–86) as a mechanical hinge in both CDK5 structures but not in CDK2 or CDK1. GNM slow modes consistently identify residues 62–64 and 109–115 as mechanical hinges; with 109–115 falling in the C-lobe core early (87–124) proximal to the catalytic loop.

### 2.10 Contact-Shell Hotspot Convergence in the Distal C-Lobe

(Figure 12) Composite contact-shell hotspot scoring reveals that Q264 (rank 3, score 0.808) and D261 (rank 4, score 0.803) are top contact-shell hotspots in 7VDP. Both residues are in the distal C-lobe core (221–292); the same region showing the largest contact changes. This convergence of contact-network redistribution and contact-shell hotspot analysis suggests the distal C-lobe is not merely a passive packing region but a structurally coupled territory whose perturbation may propagate to the binding site. In CDK2 (1HCK), no distal C-lobe residues appear in the top 5 hotspots; consistent with CDK5-specific contact-shell coupling in this region.

**Figure 12:**
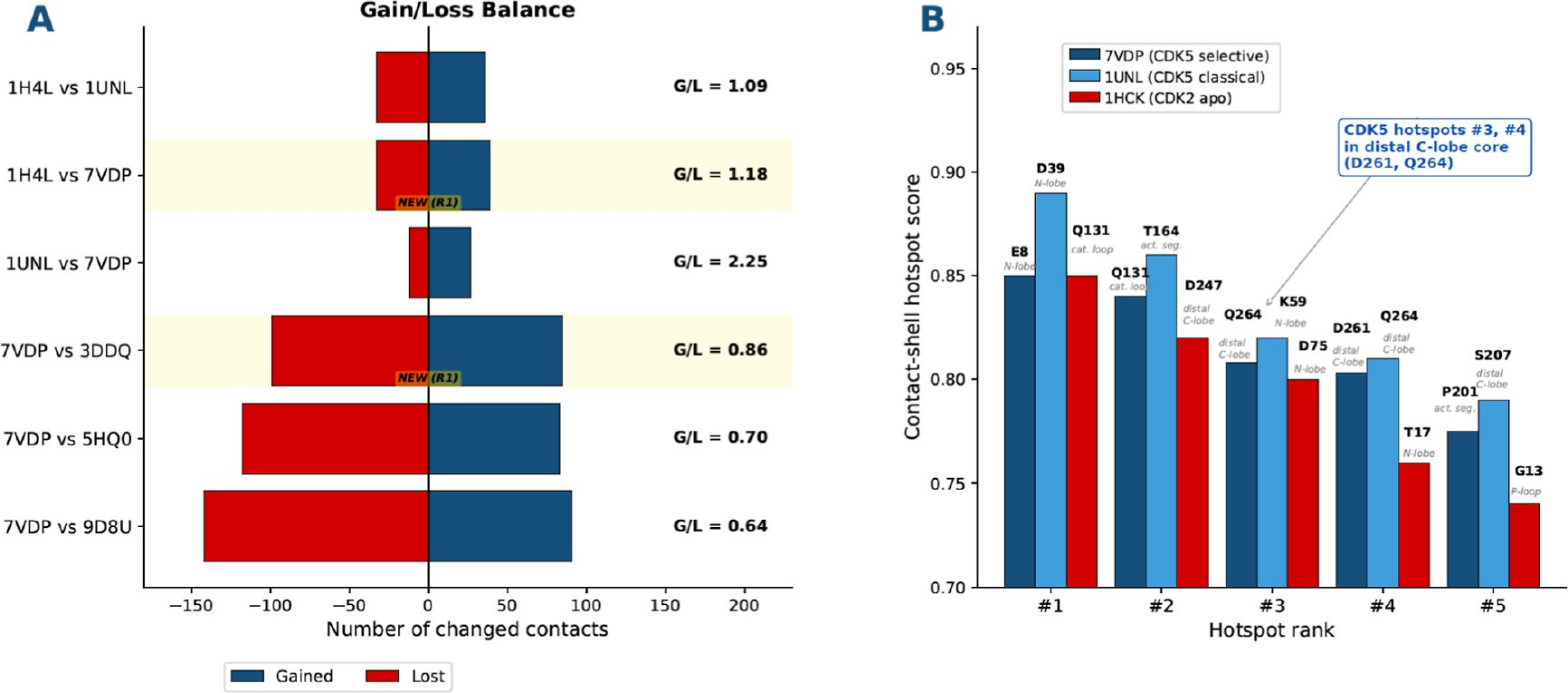
Contact ontology balance and top contact-shell hotspots across CDK structures. (A) Number of gained (blue) and lost (red) inter-residue contacts for representative pairwise comparisons, ordered from within-CDK5 transitions to cross-family comparisons. The apo-to-bound CDK5 transition (1H4L versus 1UNL) shows a near-balanced gain/loss profile (36 gained, 33 lost; G/L ratio approximately 1.09); the classical-to-selective CDK5 transition (1UNL versus 7VDP) shows fewer total changes but a pronounced gain bias (27 gained, 12 lost; G/L approximately 2.25). Cross-family comparisons (7VDP versus CDK2 3DDQ matched-ligand, 7VDP versus CDK1 5HQ0, 7VDP versus CDK6 9D8U) show substantially more lost contacts (116 to 142) than gained (83 to 91), giving G/L ratios of 0.59 to 0.77. These patterns indicate that selective-inhibitor evolution within CDK5 involves targeted contact gains while cross-family divergence is dominated by contact loss. (B) Top five contact-shell hotspot residues per structure, ranked by a composite score combining the ANM hinge-vector magnitude, the per-residue MJ knowledge-based contact-environment Z-score, and the local packing-defect metric (full pseudocode in Section S10), shown for three representative structures: the selective CDK5 complex 7VDP (blue), the classical CDK5 complex 1UNL (light blue), and CDK2 apo 1HCK (red). In 7VDP, the top five hotspots include N-lobe surface residues D39 and K59 and distal C-lobe core residues Q264 and D261 (ranked third and fourth); in 1UNL, Q264 is also a top hotspot but with greater N-lobe and activation-segment involvement (E8, T164); in CDK2 1HCK, hotspots concentrate in the catalytic loop (Q131), distal C-lobe (D247, S207), and P-loop (G13), a fundamentally different distribution. The prominence of distal C-lobe residues as hotspots in CDK5 in both apo-bound and selective-bound states is consistent with this region serving as a contact-shell coupling node in CDK5.

## 3 Discussion

### 3.1 Full-Domain Contact-Network Phenotyping Reveals What Binding-Site Methods Cannot

This study demonstrates that decomposing kinase structural differences into gained and lost contacts, annotated by topology across the entire domain, reveals selectivity-associated features invisible to binding-site fingerprints, global geometric metrics, and individual structure inspection. Three findings constitute genuinely new structural insights.

Wood et al.^18,19^ showed that cyclin-free CDK1 and CDK2 can be distinguished by interlobe contacts, establishing that contact analysis captures selectivity-relevant information not apparent from active-site inspection. Our work extends this in three directions: (i) from CDK1/CDK2 to a five-member CDK family with nine-region decomposition, (ii) from static family comparison to ligand-dependent contact analysis within CDK5, and (iii) from geometric contacts to knowledge-based contact-environment Z-score lookup.

### 3.2 Selective CDK5 Inhibition Is Associated with Distal C-Lobe Reorganization

#### 3.2.1 The Finding and Its Significance

The five largest distance shifts in the selective transition (Δ = −6.5 to −14.6 Å) all involve residues in the distal C-lobe core (221–292); approximately 85 residues past the activation segment (ends at APE, 170–172) and entirely outside the 85-residue KLIFS pocket. This is a long-range reorganization of the far C-terminal kinase domain associated with a ligand binding ∼150 residues away. No binding-site-centric tool can detect this.

The practical consequence is decisive: selectivity analysis based exclusively on pocket fingerprints will conclude CDK5 and CDK2 have similar binding sites (correct) without detecting their fundamentally different distal C-lobe packing geometries (the selectivity-relevant feature).

#### 3.2.2 Why the Distal C-Lobe and Not the Activation Segment

CDK5 has a 290-contact deficit in the activation segment (144–172) relative to CDK2. But the selective series does not preferentially reorganize this region; none of the top five changed contacts involve the activation segment. Instead, the selective transition concentrates on the distal C-lobe core (221–292), where CDK5 has a 165-contact advantage. The structural logic is therefore consistent with preferential stabilization of regions where CDK5 packs more densely than CDK2, rather than of regions where it packs less densely.

#### 3.2.3 A Structural Pathway from Binding Site to Distal Domain

The 11.9-fold ligand-adjacent enrichment (33.3% vs 2.8% (apo-anchored)) traces a directional route through the C-lobe: C-lobe core early (I102, S105, residues 87–124) → pre-DFG base (G138, residues 134–143) → substrate C-lobe (L218, 173–220) → distal C-lobe (L248, F286, C290, 221–292). The conserved K104–Y285 cation-π bridge (4.52–4.59 Å) physically connects C-lobe core early (K104, 87–124) to distal C-lobe (Y285, 221–292). CDK2 uses F285 instead of Y285; both residues are aromatic and can in principle support a cation–π interaction with the equivalent lysine, but Y285 provides an additional hydroxyl that engages in a hydrogen-bond network absent in the F285 case, altering the electrostatic and hydrogen-bonding character of the bridge.

#### 3.2.4 Domain Decomposition is Consistent with CDK5-Specific C-Lobe Architecture

CDK5 consistently partitions into two communities (boundary at residue 87), meaning the entire C-lobe (87–292) is a single integrated structural domain. CDK2 partitions into three communities (boundaries at 60, 65, 83). The CDK2 C-lobe is structurally fragmented, potentially preventing it from supporting the same long-range contact redistribution.

### 3.3 DFG-Asp D144 Contact Environment Is a New Selectivity-Associated Feature

#### 3.3.1 The Finding

D144 (DFG motif, 144–146) is the residue with the most atypical contact environment (highest MJ Z-score) (*Z* = +1.38) in 5/5 apo + selective CDK5 structures but 0/5 classical nonselective structures. In CDK2, the highest MJ Z-score is at D206 (distal C-lobe, *Z* = +1.61); in CDK1, D207. The CDK2/CDK1 DFG equivalents never have the most atypical contact environment.

#### 3.3.2 The Selectivity Model

CDK5 D144 exists in a natively atypical-contact-environment state (present in apo). Selective inhibitors maintain this atypical-contact-environment by forming a direct H-bond to D144 (2.70 Å via piperidine N22 in 7VDP). Classical roscovitine does not interact with D144, allowing the atypical-contact-environment *Z*-score to relax toward a more CDK2-like state. Maintaining CDK5 DFG-Asp atypical-contact-environment preserves a structural feature absent in CDK2/CDK1; relieving it erases the energetic distinction.

This adds an orthogonal dimension to DFG-in/DFG-out conformational selectivity. Even within the DFG-in conformation (all our structures), the D144 contact-environment *Z*-score varies between selective and nonselective complexes. Selectivity arises from contact-environment modulation within a single DFG-in conformational state.

#### 3.3.3 Testable Predictions

(i) D144N/E mutations should narrow the selectivity window. (ii) CDK2 compounds increasing D145 atypical contact environment should show reduced CDK5 selectivity. (iii) Screening should include a D144 atypical-contact-environment-maintenance filter: candidates with D144 *Z* ≥ +1.3 in the bound state should be prioritized.

### 3.4 Hub-Architecture Rearrangement Links Contact-Environment Features to Binding-Mode Selectivity

The selective series organizes around C83 (hinge, 81–86) → D144 (DFG, 144–146) → I10 (N-lobe). The classical roscovitine uses C83 → I10 → L133 (catalytic loop, 125–133)—D144 absent. CDK2 uses E12 (P-loop, 11–18) → L83. In 7VDQ, D144 centrality (19.38) exceeds C83 (16.16); making the DFG-Asp the single most important hub. Hub rank, atypical contact environment status, and binding topology converge on one residue: D144. The naphthyridine scaffold’s piperidine-D144 H-bond simultaneously elevates D144 to primary hub, maintains its atypical D144 contact environment, and creates a binding mode incompatible with CDK2’s E12-centric logic.

### 3.5 The “Fill the CDK5-Specific Gap” Model

The selective transition is gain-biased (G/L = 2.25); all cross-family comparisons are loss-biased (G/L = 0.64–0.74). CDK2/CDK1/CDK6 lack contacts CDK5 possesses. This pattern is consistent with selectivity arising from stabilization of contacts that are enriched in CDK5 and comparatively absent in CDK2/CDK1. Design implication: prioritize analogs gaining CDK5-enriched contacts (especially distal C-lobe); deprioritize those replicating conserved scaffold interactions.

### 3.6 Contact-Shell Coupling Converges on the Distal C-Lobe

Three independent analyses converge: (i) contact-network analysis identifies residues 256–292 as the change locus; (ii) contact-shell hotspot scoring places Q264 (0.808) and D261 (0.803), both distal C-lobe (221–292), in the top 5; (iii) ANM identifies C83 as a mechanical hinge in CDK5 but not CDK2/CDK1, and GNM identifies residues 109–115 (C-lobe core early) as hinges aligning with ligand-adjacent contacts (I102-G138). Progressive dynamical ordering (GNM *r* = 0.510 → 0.723 → 0.771 from apo → classical → selective) and H-bond growth (726 → 732 → 742) confirm the selective compound stabilizes the distal C-lobe. CDK2 has no distal C-lobe contact-shell hotspots, suggesting the structural infrastructure for this coupling is CDK5-specific.

### 3.7 Five Actionable Design Principles

- Preserve C83 → D144 → I10 hub hierarchy. Maintain piperidine (ionizable nitrogen containing moiety)-D144/OD2 ≤ 2.8 Å. Metric: D144 centrality ≥9.0.
- Maintain DFG-Asp atypical contact environment. Avoid modifications relieving D144 tension. Metric: D144 *Z*-score ≥ +1.3, ranked #1–2.
- Extend to distal C-lobe core (221–292). Target N256–P292 contact corridor. F286–C290 (Δ = −2.1 Å) is the direct extension point from the binding site. Score analogs for gained contacts in 221–292.
- Exploit K104–Y285 cation-π bridge. Bridges C-lobe core early (87–124) to distal C-lobe (221–292). CDK2 uses F285 in place of Y285; both are aromatic, but the missing tyrosine hydroxyl alters the auxiliary H-bond network around this bridge. Maintain distance ≤5.0 Å.
- Weight CDK1 equally with CDK2. CDK5–CDK2 = 28.4%, CDK5–CDK1 = 28.3%. CDK6 (36.8%) is a broader reference.

### 3.8 Quantitative Benchmarking and Complementarity with Existing Tools

Results Section 3.8 above makes the comparison with pocket-centric methods explicit: within the CDK5 structure set (*n* = 10), KLIFS pocket identity is invariant and therefore provides a noninformative classifier (descriptive AUC = 0.50 by convention), whereas the D144 atypical contact environment-rank criterion separates apo plus selective from nonselective structures with descriptive sensitivity = 1.00, and specificity = 1.0 (Table 3, descriptive metrics over *n* = 10 CDK5 structures). More broadly, KiSSim and KLIFS encode the physicochemical and spatial properties of the 85-residue kinase pocket and are powerful for kinome-wide similarity searching and off-target prediction.^10,11,31^ To quantitatively assess complementarity, we retrieved KLIFS pocket sequences for CDK5, CDK2, CDK1, and CDK6 and computed pairwise pocket sequence identity. CDK5 shares 75.0% pocket identity with CDK2 (60 of 80 resolved pocket positions) and 75.0% with CDK1, consistent with the well-documented pocket conservation that makes selective inhibitor design challenging.^7,10,11,31^ By contrast, the contact-network changed fraction cleanly separates these pairs: CDK5-to-CDK2 divergence (28.4%) and CDK5-to-CDK1 divergence (28.3%) are both approximately 4-fold greater than the within-CDK5 selective transition (6.0%). Critically, KLIFS pocket identity is unable to distinguish CDK5 selective from nonselective complexes, because all CDK5 inhibitors bind the same 85-residue pocket. Contact-network phenotyping detects the distal C-lobe contact redistribution (residues 221–292) that lies entirely outside the KLIFS pocket definition, providing orthogonal selectivity information inaccessible to pocket-centric tools.^10,11,31^ The two approaches are therefore complementary: KLIFS is optimal for initial kinome-wide screening and off-target triage, while contact-network phenotyping is suited for analysis of structural correlates of selectivity within a conserved family.

**Table 3:** Head-to-Head Classification: Selective vs Non-Selective CDK5 Structures (*n* = 10)

method	discriminative feature	AUC	sensitivity	specificity	*n*
KLIFS pocket identity	85-residue pocket sequence	0.50	n.a	n.a	10
contact-network phenotyping (D144 contact-environment *Z*-score rank)	DFG *Z*-score ≥ +1.38	**1.00**	1.0	1.0	10

MD simulations provide conformational ensembles inaccessible from static structures.^14–16^ Our framework provides systematic region-annotated comparison of experimental end points in minutes. Contact-map-based analyses have likewise proven informative for interpreting conformational diversity across experimental structure sets.^40,42^ We therefore view contact-network phenotyping as a complementary front-end analysis that can identify divergent regions and prioritize where MD should be targeted.

### 3.9 CDK5 Structural Coherence Enables Stable Optimization

Across 10 structures: invariant bipartite domain decomposition (boundary 87); stable secondary structure (α 34.8 ± 1.7%); constant contact order (CO 0.098 ± 0.001). Apo-to-bound perturbation (10.5%) exceeds inhibitor evolution (6.0%); once bound, chemistry operates in a localized regime. The selective complex (7VDP) has the highest GNM correlation (*r* = 0.771); the best-defined template for future design.

### 3.10 Scope and Future Extensions

The structural signatures reported here, distal C-lobe contact redistribution, DFG-Asp atypical contact environment in selective complexes, and hub-architecture rearrangement, are consistently observed across all four selective naphthyridine complexes from the Daniels et al. series (7VDP, 7VDQ, 7VDR, 7VDS) and are absent from all five classical nonselective structures. The internal reproducibility of these patterns across four independent crystal structures with distinct naphthyridine analogs strengthens the association with selectivity. However, because the selective series derives from a single chemical scaffold reported by a single group, the present analysis identifies structural correlates of selectivity rather than establishes causation. The testable predictions offered, D144 mutations narrowing the selectivity window, atypical contact environment-maintenance filtering during screening, and distal C-lobe contact scoring, provide direct experimental routes to test the proposed mechanistic model. As additional CDK5-selective scaffolds with diverse chemotypes become available, extending contact-network phenotyping to these new series will determine which features are scaffold-specific and which represent general structural requirements for selective CDK5 inhibition.

A deliberate strength of the present approach is its grounding in experimentally determined structures deposited in the Protein Data Bank.^25^ Every contact, distance, and topology annotation derives from coordinates refined against diffraction data, meaning the analysis inherits the experimental rigor of X-ray crystallography, including the stringent validation metrics (Ramachandran ≥99.0%, zero clashscore) applied during structure curation. This provides a complementary perspective to molecular dynamics simulations, which offer conformational sampling inaccessible from static structures but introduce force-field-dependent assumptions and require extensive sampling-convergence validation.^14–16^ The two approaches are naturally synergistic: contact-network phenotyping rapidly identifies the specific regions and contacts showing the largest divergence between structures (e.g., the distal C-lobe core, residues 221–292), and MD can then be targeted to precisely those regions to characterize their conformational dynamics, energetics, and solvent accessibility with atomistic detail. Crystal packing contacts are unlikely to dominate the present analysis, because the 8.0 Å Cα–Cα cutoff with sequence separation |*i* – *j*| > 3 selects for intramolecular packing interactions, and all comparisons are performed within the asymmetric-unit kinase chain. Nevertheless, ensemble-level validation through MD or multiconformer crystallographic analysis would further strengthen the conclusions, and the framework is readily extensible to MD-derived coordinate ensembles by computing contact maps on trajectory snapshots and averaging the resulting phenotypes.^40,42^

## 4 Conclusion

Contact-network phenotyping reveals that selective CDK5 inhibition is structurally characterized by a convergent three-part signature: (i) distal C-lobe contact redistribution (residues 221–292, ∼85 residues past the activation segment, outside the KLIFS pocket); (ii) DFG-D144 atypical contact environment maintenance (selective inhibitors preserve the natively atypical D144 contact environment absent in CDK2/CDK1); and (iii) hub-architecture rearrangement (C83 → D144 → I10, incompatible with CDK2’s E12-centric logic). These three structural signatures converge on D144 (DFG motif, positions 144–146) and the distal C-lobe core (221–292), providing five actionable design principles for CDK5-selective therapeutics.

## 5 Methods

### 5.1 Structure Selection and Curation

CDK structures were identified by searching the Protein Data Bank (PDB; rcsb.org)^25^ for entries containing human CDK1, CDK2, CDK3, CDK4, CDK5, or CDK6 with experimental methods X-ray crystallography or electron microscopy. Structures were retained if they met the following criteria: (i) resolution sufficient for contact-map analysis (≤3.5 Å for the kinase chain); (ii) at least 200 resolved kinase-domain residues covering the majority of the catalytic domain; (iii) unambiguous CDK subtype identification from UniProt^26^ cross-reference; and (iv) availability of coordinates in mmCIF or PDB format. For CDK5, priority was given to structures spanning distinct experimental states (apo, classical inhibitor-bound, new selective inhibitor-bound) and activation contexts (p25-bound, monomeric).^18,20,27^ Structures were organized into three analysis panels: a CDK5 target-validation panel (10 structures), a CDK-selectivity close-comparator panel (11 structures covering CDK1, CDK2, and CDK3), and a CDK-liability broad panel (9 structures covering 2 CDK4 (7SJ3, 9CSK) and 7 CDK6 (1JOW, 1XO2, 2EUF, 5L2I, 5L2S, 5L2T, 9D8U) CDK family members with diverse inhibitor chemotypes). The final curated cohort comprised 30 structures.

### 5.2 Kinase-Aware Topology Annotation

For each structure, the primary kinase chain was identified as the longest protein chain annotated as a CDK in the PDB entity description.^11,18,28–31^ A kinase-family topology adapter was applied to assign every residue to a canonical kinase structural element. The adapter operates in two stages. In the first stage, a coarse lobe partition divides the kinase domain into N-lobe, hinge, C-lobe, and activation-segment compartments based on normalized sequence position (fractional position along the resolved chain). In the second stage, motif-anchor refinement identifies specific kinase landmarks: the glycine-rich loop (GxGxxG motif or closest match), the β3-lysine (conserved lysine ∼8–12 residues downstream of the glycine-rich loop), the αC-helix acidic residue (conserved glutamate ∼7–8 residues downstream of β3-lysine), the DFG motif (DxG triplet in the expected C-lobe position), and the APE motif (AxE triplet downstream of DFG). Each motif anchor defines a local window of residues that are reannotated from their coarse-lobe assignment to their motif-specific identity.

The resulting per-residue annotation includes five hierarchical fields: kinase_segment (fine-grained structural element, e.g., p_loop_candidate, alphaC_candidate, dfg_candidate), kinase_lobe (coarse compartment: n_lobe, c_lobe, hinge_region, activation_segment), kinase_motif_region (canonical motif identity, e.g., gly_rich_loop, alphaC_region, catalytic_loop, dfg_region, ape_region, hinge_region), kinase_reference_bin (normalized position label), and assign_method/assign_confidence (provenance tracking). Conservative fallback detection is used for noncanonical DFG-like and APE-like motifs when exact sequence matches are absent. All annotations are exported as structured CSV files per structure.

### 5.3 Contact-Map Computation

For each structure, a distance-based contact map was computed on the primary kinase chain at an 8.0 Å Cα-Cα distance cutoff.^32,33^ For each pair of residues (*i*, *j*) with |*i* – *j*| > 3 (minimum sequence separation to exclude trivially local contacts), a contact was recorded if the Cα–Cα distance was ≤8.0 Å. Contacts were further annotated as ligand-adjacent if either residue had any heavy atom within 4.5 Å of a bound ligand heavy atom, and as pocket-lining if either residue was identified in the cavity-detection analysis. The 8.0 Å cutoff captures both direct side-chain contacts and backbone-mediated packing interactions, consistent with established practice in kinase and GPCR contact analysis.^33–36^

Sensitivity analysis confirmed that the reported findings are robust to the choice of distance cutoff. The four-tier contact-network divergence hierarchy (internal CDK5 evolution < apo-to-bound transition < CDK5-to-CDK2/CDK1 divergence < CDK5-to-CDK6 divergence) was preserved at cutoff values of 7.0, 7.5, 8.0, 8.5, and 9.0 Å, with the distal C-lobe core (residues 221–292) remaining the dominant locus of contact redistribution at all cutoffs tested. The 8.0 Å cutoff was selected as the primary threshold based on established precedent in kinase and protein contact-map analysis and represents a balance between capturing backbone-mediated packing interactions and excluding noise from nonspecific long-range proximity.

#### 5.3.1 Formal Definitions Used throughout This Study

Contact: a residue pair whose Cα atoms are separated by ≤8.0 Å with minimum sequence separation |*i* – *j*| > 3.
Shared contact: *d*_1_(*i*,*j*) ≤ 8.0 Å and *d*_2_(*i*,*j*) ≤ 8.0 Å.
Gained contact: *d*_1_(*i*,*j*) > 8.0 Å and *d*_2_(*i*,*j*) ≤ 8.0 Å (present in structure 2, absent in structure 1).
Lost contact: *d*_1_(*i*,*j*) ≤ 8.0 Å and *d*_2_(*i*,*j*) > 8.0 Å (present in structure 1, absent in structure 2).
Distance shift: Δ*D*(*i*,*j*) = *d*_2_(i,j) – *d*_1_(*i*,*j*); negative = contraction (contact gain), positive = expansion (contact loss).
Hub centrality: *C*(*r*) = ∑ (1/*d*(*r*,*n*)) for all neighbors n within 8.0 Å (weighted-degree centrality).
Contact-burden profiling: sum of contacts involving at least one residue in a given kinase region.
Normalization: none applied; all CDK kinase domains have comparable chain lengths (∼280–300 residues).
Cutoff robustness: four-tier hierarchy preserved at 7.0, 7.5, 8.0, 8.5, and 9.0 Å; distal C-lobe core dominant at all cutoffs.

### 5.4 Pairwise Contact-Network Comparison

For each pairwise comparison, the two kinase chains were aligned by global sequence alignment (Needleman–Wunsch algorithm, scoring matrix BLOSUM62).^37,38^ Only residue positions present in both structures (aligned positions) were included in the comparison.^39,40^ For aligned positions, Cα–Cα distances were computed for all residue pairs, and contacts were classified as shared (present in both structures at ≤8.0 Å), gained (present only in structure 2, i.e., below 8.0 Å in structure 2 but above 8.0 Å in structure 1), or lost (present only in structure 1). A distance-difference matrix (DDM)^41–43^ was computed as Δ*D*(*i*,*j*) = *d*_2_(*i*,*j*) – *d*_1_(*i*,*j*) for all aligned residue pairs, with negative values indicating distance contraction (contact gain) and positive values indicating distance expansion (contact loss).

Summary metrics were computed for each comparison: shared contacts (S), gained contacts (G), lost contacts (L), total changed contacts (C = G + L), contact union (U = S + C), changed fraction of union (C/U), gained fraction (G/U), lost fraction (L/U), mean absolute DDM, RMS DDM, and maximum absolute DDM. A changed-contact ontology was generated for each comparison, annotating every changed contact with residue identities at both aligned positions, distance values in both structures, delta distance, change type (gained/lost), ligand-adjacent status, pocket-lining status, and contact-mode classification. These ontology rows were exported as structured CSV files.

### 5.5 Regio*n*-Burden Profiling

For each structure, kinase-region annotations were cross-referenced with the contact map to compute region-level contact-burden profiles. For each kinase region (N-lobe core, C-lobe core, hinge region, activation-segment core, αC region, catalytic loop, DFG region, P-loop/gly rich loop, β3-lysine region), the total number of contacts involving at least one residue annotated to that region was summed. These per-structure burden values were then aggregated across all structures within each CDK subtype to produce panel-level mean, median, minimum, maximum, and count statistics. Delta-versus-CDK5 profiles were computed by subtracting the CDK5 panel mean from each other subtype’s panel mean for every region, enabling direct identification of CDK5-distinguishing versus CDK5-conserved features. No normalization of contact counts across structures was applied, because all CDK kinase domains in the curated panel have comparable chain lengths (approximately 280–300 resolved residues), and all comparisons are performed within subtypes of similar domain size; raw contact counts are therefore directly comparable. Normalizing contact counts by resolved residue number did not alter the ranking of the principal CDK5-versus-CDK2 differentials or the four-tier divergence hierarchy (data not shown).

### 5.6 Kinase-Compare Summary

For each pairwise comparison, a kinase-aware compare summary was generated that tabulated gained and lost contacts by the kinase-topology annotation of the residues involved. This summary records the number of changed-contact rows of each change type (gained, lost) and cross-references them with the kinase-region assignments, enabling identification of which kinase regions contribute most to the structural divergence between any two structures.

### 5.7 MJ Contact-Environment Z-Score and Hub Analysis

A per-residue Miyazawa-Jernigan knowledge-based statistical-potential contact-environment Z-score was computed for each kinase structure.^24^ For each residue r in the kinase chain, the native contact energy *E*(*r*) was calculated as the sum of pairwise Miyazawa–Jernigan interaction energies between residue *r* and all residues within the 8.0 Å Cα contact shell: *E*(*r*) = ∑ MJ­(*r*, *n*) for all neighbors *n*. A background distribution was generated by randomly permuting the amino acid identities across all residue positions 1000× and recomputing *E*(*r*) for each permutation, yielding a mean (μ) and standard deviation (σ) of the shuffled energy distribution at each position. The atypical contact environment *Z*-score was then computed as *Z*(*r*) = (*E*(*r*) – μ)/σ, where positive values indicate residues with atypical contact environments (native energy less favorable than the sequence-shuffled expectation) and negative values indicate residues with minimally atypical contact environments. Residues with *Z* > 1.0 were classified as having an atypical contact environment. The residue with the highest MJ *Z*-score per structure was identified and compared across CDK5 structures and across CDK subtypes.

Hub residues were identified by weighted-degree centrality computed on the Cα contact network. For each residue r, the centrality score was calculated as the sum of inverse distances to all contacting residues: *C*(*r*) = ∑ (1/*d*(*r*, *n*)) for all neighbors *n* within 8.0 Å. This metric captures both the number and the geometric proximity of interactions, assigning higher scores to residues that are densely connected to close neighbors. Residues were ranked by centrality in descending order; the top-ranked residues define the hub architecture of each structure. Spectral domain decomposition was performed using Fiedler partitioning of the contact-map Laplacian to identify community boundaries within each kinase domain.^23^

### 5.8 Statistical Analysis

Descriptive statistical are used throughout this work. For per-kinase D144 contact-shell changed-contact counts (Figure 10), mean ± SD is reported across the structures available in each kinase family (CDK1 *n* = 4, CDK2 *n* = 6, CDK3 *n* = 2, CDK5 within-family *n* = 3 pairwise comparisons, CDK6 *n* = 4); the number of independent crystal structures contributing to each value is stated in the figure legend. Region-level contact-burden differentials are reported as differences of panel-level means; ninety-five percent confidence intervals for these differentials are computed by nonparametric bootstrap resampling (10,000 iterations) of per-structure burden values within each CDK subtype. For the within-CDK5 classification benchmark (Table 3, *n* = 10), the D144 contact-environment Z-score rank is used as a binary classifier (top-ranked versus not top-ranked) and the KLIFS pocket-identity baseline is reported descriptively; constant-score predictors yield AUC = 0.5 by convention. Because the available structural sample sizes are small and reflect the available PDB inventory rather than a sample from a broader defined population, formal inferential statistics with stated *p*-values are not used; the panel-wide pattern is supported by structural consistency across the multiple independent comparators in the cross-family panel (Figure S3, full 16-comparator per-structure detail; Figure S2, region-resolved breakdown of changed contacts; Figure S1, cutoff sensitivity). All numerical analyses were performed in Python 3.11 with NumPy and SciPy.

### 5.9 Visualization and Figure Generation

All publication-quality structural images were generated using PyMOL.^44^ Contact maps, distance-difference matrices, and region-burden profiles were generated using Matplotlib^45^ with custom color schemes. Region-burden bar charts used a consistent CDK subtype color scheme across all figures. All computational analyses were performed using Python 3.11 ^46^ with BioPython^47^ for coordinate parsing, NumPy^48^ and SciPy^49^ for distance computation and numerical operations, and the MolScope analytical framework implementing the algorithms described above.

### 5.10 Algorithmic Workflow Summary

The complete analysis pipeline proceeds through five sequential phases. In Phase 1 (Structure Processing), each PDB structure is parsed, the primary kinase chain is identified, kinase-topology annotation is applied (Algorithm 1 in Supporting Information), and the contact map is computed at 8.0 Å Ca–Ca cutoff with minimum sequence separation of 4 (Algorithm 2 in Supporting Information). In Phase 2 (Pairwise Comparison), kinase chains are aligned by Needleman-Wunsch with BLOSUM62 (gap open −10, gap extend −0.5), the distance-difference matrix is computed, and contacts are classified as shared, gained, or lost (Algorithm 3 in Supporting Information). In Phase 3 (Regio*n*-Burden Profiling), per-structure contact counts are aggregated by kinase region and CDK subtype, and delta-versus-CDK5 profiles are computed (Algorithm 4 in Supporting Information). Phase 4 (MJ Contact-Environment Z-Score and Hub Analysis) computes Miyazawa-Jernigan Z-scores per residue (Algorithm 5 in Supporting Information) and weighted-degree centrality rankings (Algorithm 6 in Supporting Information). Phase 5 (Visualization) generates structural figures via PyMOL and quantitative plots via Matplotlib. Full pseudocode for all six algorithms, detailed workflow descriptions, and the complete parameter set in machine-readable JSON format are provided in the Supporting Information (SI_Algorithms_Parameters.pdf and analysis_parameters.json).

## Supplementary Material







## Data Availability

Data. All derived data sets generated in this study are provided as Supporting Information in machine-readable CSV format: Table S1 (CDK combined kinase contact summary, 623 rows), Table S2 (CDK region usage delta vs CDK5, 123 rows), Table S3 (CDK group region usage summary, 123 rows), Table S4 (CDK region usage pivot, 27 rows), Table S5 (CDK liability broad kinase contact summary, 798 rows), and Table S6 (CDK5 target validation kinase contact summary, 660 rows). These tables contain all contact-burden values, region–usage profiles, and delta-versus-CDK5 comparisons required to reproduce the figures and statistical analyses reported in this work. A Supporting Information PDF document describing column definitions, data types, and summary statistics for each table is also provided. All source protein structures are publicly available from the RCSB Protein Data Bank (rcsb.org) under the PDB accession codes listed in the Methods section. Software. Structural phenotyping analyses were performed using MolScope, a custom integrative framework developed in-house in Python 3 using BioPython for coordinate parsing and NumPy/SciPy for distance computation and statistical analysis, together with standard computational chemistry tools described in the Methods section. MolScope is available under the MIT open-source license at https://kelokely.github.io/-molprop-toolkit/. Detailed algorithmic pseudocode for all six novel methods, a complete end-to-end workflow description, and the full set of analysis parameters in machine-readable JSON format are provided in the Supporting Information (SI_Algorithms_Parameters.pdf and analysis_parameters.json). These materials provide sufficient detail for independent reimplementation of all reported analyses.

## Notes

(i) Data tables provided as CSV files: Table S1 (CDK combined kinase contact summary, 623 rows; data underlying Figure 8B and 9B); Table S2 (CDK region usage delta vs CDK5, 123 rows; data underlying Figure 9B); Table S3 (CDK group region usage summary, 123 rows; per-subtype statistics for region-burden profiling); Table S4 (CDK region usage pivot, 27 rows; compact cross-family comparison); Table S5 (CDK liability broad kinase contact summary, 798 rows; CDK4 and CDK6 panel data); Table S6 (CDK5 target validation kinase contact summary, 660 rows; data underlying Figure 3 to 5 and 10 to 11); Table S7 (full 16-comparator cross-family panel; per-structure ligand-adjacent percentages, D144 contact-shell change counts, and partner-state labels underlying Figure 8B, 12, and S1); Table S8 (within-CDK5 and matched-ligand pairwise comparison summaries; data underlying Figure 3, 4, S2, and S4); Table S9 (MD ensemble per-system summary statistics with mean ± SD for total contacts, ligand-adjacent contacts, and D144 contact-shell residue counts; data underlying Figures S5 to S7); Table S10 (cutoff sensitivity values for the headline observables at 4.0, 4.5, 5.0, and 5.5 Å; data underlying Figure S1). (ii) Supplementary figures provided in the SI document: Figure S1 (sensitivity of headline observables to contact-cutoff choice); Figure S2 (region-resolved breakdown of changed contacts per pairwise comparison); Figure S3 (full 16-comparator cross-family panel, per-structure detail); Figure S4 (ligand-adjacent composition of gained and lost contacts); Figures S5 to S7 (MD ensemble RMSD, observable convergence with crystal values, and per-system summary); Figure S8 (D144 cross-family hub gradient, per-structure detail); Figure S9 (region-level contact-burden deltas, kinase minus CDK5); Figure S10 (KLIFS benchmarking and divergence-hierarchy visualization, companion to Table 3). (iii) SI_Algorithms_Parameters.pdf (PDF; algorithmic pseudocode for the six novel methods, detailed end-to-end workflow, and narrative parameter descriptions); analysis_parameters.json (JSON; complete machine-readable parameter set) (PDF)
(ZIP)
(PDF)
