# Supplementary material for: Contact-Network Phenotyping of the CDK Family Reveals Selective Distal C‑Lobe Contact Redistribution by Modern CDK5 Inhibitors and a Quantitative Selectivity Landscape against CDK2 and CDK1
Source: J Chem Inf Model. 2026 May 29;66(12):7276–95. doi: 10.1021/acs.jcim.6c00886 (PMC13292211; doi:10.1021/acs.jcim.6c00886)
Supplement: Supplementary file 1 [file ci6c00886_si_001.pdf]

Supporting Information

Contact-Network Phenotyping of the CDK Family Reveals Selective Distal C-Lobe Contact Redistribution by Modern CDK5 Inhibitors and a Quantitative Selectivity Landscape Against CDK2 and CDK1

*Manal A. Nael, Laxman M. Alakonda, Khaled M. Elokely**

***Corresponding author.** Email: kelokely@uwyo.edu; Phone: (307) 766-6136.

This Supporting Information accompanies the main manuscript and provides: (i) ten supplementary data tables (Tables S1 to S10) containing the per-structure contact-burden, region-usage, ligand-adjacency, cross-family, MD-ensemble, and cutoff-sensitivity datasets underlying the reported analyses; (ii) ten supplementary figures (Figures S1 to S10) providing per-structure detail and robustness analyses for the main-text figures; (iii) algorithmic pseudo-code and the complete parameter set in machine-readable JSON format; (iv) MD ensemble protocols. All data tables are provided as separate CSV files; all figures are provided as separate TIFF and PDF files in the SI archive.

Contents

S1. Index of Supplementary Data Tables (S1 to S10)

S2. Description of New Supplementary Tables (S7 to S10)

S3. Supplementary Figure Legends (S1 to S10)

S4. Cutoff and Parameter Sensitivity Analysis

S5. Cross-Family Panel and Partner-State Considerations

S6. Region-Burden Profiling Details

S7. D144 Cross-Family Contact-Shell Hub Analysis

S8. KLIFS Benchmarking Detail

S9. Molecular Dynamics Protocols and Robustness Check

S10. Algorithmic Pseudo-Code and Parameter Set

S1. Index of Supplementary Data Tables

All supplementary data tables are provided as machine-readable CSV files in the SI archive.

Table	Filename	Rows	Description
S1	TableS1_CDK_combined_kinase_contact_summary.csv	623	CDK combined kinase contact summary; data underlying Figures 8B and 9B.
S2	TableS2_CDK_region_usage_delta_vs_CDK5.csv	123	CDK region usage delta versus CDK5; data underlying Figure 9B.
S3	TableS3_CDK_group_region_usage_summary.csv	123	CDK group region usage summary; per-subtype statistics for region-burden profiling.
S4	TableS4_CDK_region_usage_pivot.csv	27	CDK region usage pivot; compact cross-family comparison.
S5	TableS5_CDK_liability_broad_kinase_contact_summary.csv	798	CDK liability broad kinase contact summary; CDK4 and CDK6 panel data.
S6	TableS6_CDK5_target_validation_kinase_contact_summary.csv	660	CDK5 target validation kinase contact summary; data underlying Figures 3 to 5 and 10 to 11.
S7	TableS7_full_16_comparator_cross_family_panel.csv	16	Full 16-comparator cross-family panel; per-structure ligand-adjacent percentages, D144 contact-shell change counts, and partner-state labels.
S8	TableS8_within_CDK5_and_matched_ligand_pair_summaries.csv	7	Within-CDK5 and matched-ligand pairwise comparison summaries; total changed, gained, lost, ligand-adjacent counts, D144 changed-contact counts.
S9	TableS9_MD_ensemble_per_system_summary.csv	5	MD ensemble per-system summary statistics with mean ± SD across 60 ensemble snapshots per system for total contacts, ligand-adjacent contacts, D144 contact-shell residue counts, and per-region contact counts.
S10	TableS10_cutoff_sensitivity.csv	4	Cutoff sensitivity values at 4.0, 4.5, 5.0, and 5.5 Å for the headline apo-anchored ligand-adjacent enrichment ratio, within-CDK5 and cross-family changed-contact fractions, and per-kinase D144 contact-shell change counts.

S2. Description of New Supplementary Tables

Table S1. CDK Combined Kinase Contact Summary

File: Table_1_cdk_combined_kinase_contact_summary_long.csv | **Rows:** 623 | **Columns:** 10

Description. Per-structure kinase contact-burden summary combining all three analysis panels (CDK5 target validation, CDK selectivity close-comparator, CDK liability broad). Each row reports the total number of contact-side counts for a specific kinase structural feature (kinase segment or kinase lobe) in a given PDB structure. This table enables direct comparison of contact-network properties across all 28 CDK structures analyzed in the study.

Column Definitions

Column	Type	Non-null	Example
panel_name	Text	623	cdk5_target_validation
pdb_id	Text	623	1H4L
target_group	Text	623	CDK5
target_name	Text	623	CDK5
structure_name	Text	623	1H4L
summary_group	Text	623	kinase_segment
feature_name	Text	623	activation_segment_core
feature_value	Numeric (integer)	623	499
notes	Text	623	contact-side counts by kinase segment
source_panel	Text	623	cdk5

Key Statistics

pdb_id: 28 unique values
target_group (5 unique): CDK1, CDK2, CDK4, CDK5, CDK6
target_name (5 unique): CDK1, CDK2, CDK4, CDK5, CDK6
summary_group (3 unique): kinase_lobe, kinase_motif_region, kinase_segment
feature_name: 25 unique values

feature_value: range [10.0, 9097.0], mean 583.5 (n=623)

Table S2. CDK Region Usage Delta vs. CDK5

File: Table_2_cdk_region_usage_delta_vs_cdk5.csv | **Rows:** 123 | **Columns:** 6

Description. Region-level contact-burden differences between each CDK subtype and CDK5. For each kinase region (e.g., hinge, C-lobe core, activation segment), the panel-mean contact burden of each CDK subtype is compared to the CDK5 panel mean. Positive delta values indicate higher burden in the non-CDK5 subtype; negative values indicate CDK5-enriched burden. This table directly supports the region-burden profiling analysis (Figure 9) and the identification of CDK5-distinguishing features such as the fivefold hinge differential and the 165-contact C-lobe advantage.

Column Definitions

Column	Type	Non-null	Example
target_group	Text	123	CDK1
summary_group	Text	123	kinase_lobe
feature_name	Text	123	activation_segment
mean_feature_value	Numeric (float)	123	566.0
cdk5_mean	Numeric (float)	105	533.7
delta_vs_cdk5	Numeric (float)	105	32.299999999999955

Key Statistics

target_group (5 unique): CDK1, CDK2, CDK4, CDK5, CDK6
summary_group (3 unique): kinase_lobe, kinase_motif_region, kinase_segment
feature_name: 25 unique values

mean_feature_value: range [22.8, 5078.3], mean 587.9 (n=123)
cdk5_mean: range [28.8, 5078.3], mean 773.2 (n=105)
delta_vs_cdk5: range [-2681.6, 290.1], mean -97.9 (n=105)

Table S3. CDK Group Region Usage Summary

File: Table_3_cdk_group_region_usage_summary.csv | **Rows:** 123 | **Columns:** 9

Description. Descriptive statistics (mean, median, min, max, count) for contact-burden values across kinase regions, aggregated by CDK subtype. Each row represents a specific kinase region feature for a given CDK group, providing the statistical distribution of contact-burden values across all structures of that subtype. This table provides the underlying data for the CDK-family region-burden comparison.

Column Definitions

Column	Type	Non-null	Example
target_group	Text	123	CDK1
target_name	Text	123	CDK1
summary_group	Text	123	kinase_lobe
feature_name	Text	123	activation_segment
mean	Numeric (float)	123	566.0
median	Numeric (float)	123	567.5
min	Numeric (integer)	123	551
max	Numeric (integer)	123	578
count	Numeric (integer)	123	4

Key Statistics

target_group (5 unique): CDK1, CDK2, CDK4, CDK5, CDK6
target_name (5 unique): CDK1, CDK2, CDK4, CDK5, CDK6
summary_group (3 unique): kinase_lobe, kinase_motif_region, kinase_segment
feature_name: 25 unique values

mean: range [22.8, 5078.3], mean 587.9 (n=123)

Table S4. CDK Region Usage Pivot

File: Table_4_cdk_region_usage_pivot.csv | **Rows:** 27 | **Columns:** 7

Description. Pivot-format table showing mean contact-burden values for each kinase region (rows) across all five CDK subtypes (columns: CDK1, CDK2, CDK4, CDK5, CDK6). This compact format enables direct visual comparison of region-level contact properties across the CDK family, supporting the hierarchical selectivity landscape analysis.

Column Definitions

Column	Type	Non-null	Example
summary_group	Text	27	kinase_lobe
feature_name	Text	27	activation_segment
CDK1	Numeric (float)	21	566.0
CDK2	Numeric (float)	27	823.8
CDK4	Numeric (float)	27	750.0
CDK5	Numeric (float)	21	533.7
CDK6	Numeric (float)	27	711.0

Key Statistics

summary_group (3 unique): kinase_lobe, kinase_motif_region, kinase_segment
feature_name: 25 unique values

Table S5. CDK Liability Broad Kinase Contact Summary

File: Table_5_cdk_liability_broad_kinase_contact_summary_long.csv | **Rows:** 798 | **Columns:** 9

Description. Per-structure kinase contact-burden summary for the CDK-liability broad panel (9 structures covering CDK4 and CDK6 with diverse inhibitor chemotypes). Each row reports the total contact-side counts for a specific kinase structural feature in a given PDB structure. This panel provides the broader CDK-family context for assessing off-target liability.

Column Definitions

Column	Type	Non-null	Example
panel_name	Text	798	cdk_liability_broad
pdb_id	Text	798	7SJ3
target_group	Text	798	CDK4
target_name	Text	798	CDK4
structure_name	Text	798	7SJ3
summary_group	Text	798	kinase_segment
feature_name	Text	798	activation_loop_candidate
feature_value	Numeric (integer)	798	188
notes	Text	798	contact-side counts by kinase segment

Key Statistics

pdb_id (9 unique): 1JOW, 1XO2, 2EUF, 5L2I, 5L2S, 5L2T, 7SJ3, 9CSK, 9D8U

target_group (2 unique): CDK4, CDK6

target_name (2 unique): CDK4, CDK6

summary_group (6 unique): contact_context, kinase_lobe, kinase_lobe_pair, kinase_motif_region, kinase_segment, kinase_segment_pair

feature_name: 111 unique values

feature_value: range [1.0, 7358.0], mean 181.2 (n=798)

Table S6. CDK5 Target Validation Kinase Contact Summary

File: Table_6_cdk5_target_validation_kinase_contact_summary_long.csv | **Rows:** 660 | **Columns:** 9

Description. Per-structure kinase contact-burden summary for the CDK5 target-validation panel (10 structures: 1 apo, 5 classical inhibitor-bound, 4 selective naphthyridine-bound). Each row reports the total contact-side counts for a specific kinase structural feature in a given PDB structure. This table supports the internal consistency assessment and the identification of contact-network changes between classical and selective CDK5 inhibitor series.

Column Definitions

Column	Type	Non-null	Example
panel_name	Text	660	cdk5_target_validation
pdb_id	Text	660	1H4L
target_group	Text	660	CDK5
target_name	Text	660	CDK5
structure_name	Text	660	1H4L
summary_group	Text	660	kinase_segment
feature_name	Text	660	activation_segment_core
feature_value	Numeric (integer)	660	499
notes	Text	660	contact-side counts by kinase segment

Key Statistics

pdb_id (10 unique): 1H4L, 1UNG, 1UNH, 1UNL, 3O0G, 4AU8, 7VDP, 7VDQ, 7VDR, 7VDS
target_group (1 unique): CDK5
target_name (1 unique): CDK5
summary_group (6 unique): contact_context, kinase_lobe, kinase_lobe_pair, kinase_motif_region, kinase_segment, kinase_segment_pair
feature_name: 77 unique values

feature_value: range [1.0, 5573.0], mean 422.6 (n=660)

Table S7. Full 16-comparator cross-family panel.

Table S7 provides the per-structure data underlying Figure 8B (cross-family contact-network divergence), Figure 12A (D144 cross-family hub gradient), and Figure S3 (per-structure detail of the cross-family panel). Each row represents one pairwise comparison between the modern selective CDK5 complex (PDB 7VDP) and one cross-family comparator structure. Columns: comparator (PDB code of the cross-family structure); kinase (CDK1, CDK2, CDK3, CDK4, or CDK6); partner (biological assembly partner, e.g., cyclin, p25, CKS, or 'monomeric'); total (total number of changed contacts between 7VDP and the comparator at the 8.0 Å Cα cutoff); gained, lost (component counts of changed contacts); g_LA_pct (percentage of gained

contacts that are ligand-adjacent in the comparator structure, within 4.5 Å of any ligand heavy atom); asp144_total (number of D144 contact-shell changed contacts in the comparison, used for the non-circular cross-family hub analysis in Figure 10); partner_state (categorical label used for the partner-state stratification in Figure 12B: partner-bound, monomeric apo, or monomeric with ligand).

Table S8. Within-CDK5 and matched-ligand pairwise comparison summaries.

Table S8 provides the seven pairwise comparison summary rows underlying the within-CDK5 and matched-ligand analyses in the main text. The three within-CDK5 rows (1H4L to 1UNL apo-to-non-selective; 1H4L to 7VDP apo-to-selective; 1UNL to 7VDP non-selective-to-selective) supply the headline apo-anchored ligand-adjacent enrichment (11.9-fold) reported in Figure 4B. The matched-ligand row (1UNL to 3DDQ) and the three ligand-mismatched cross-family rows (7VDP to 9GP3, 7VDP to 5HQ0, 7VDP to 5L2I) supply the ligand-identity-versus-kinase-identity decomposition discussed in Section 6 of the main text. Columns: pair (pair identifier); description (biological description of the two endpoints); total_changed, gained, lost (contact ontology counts); gained_LA, gained_LA_pct (ligand-adjacent gained contact count and percentage); lost_LA, lost_LA_pct (ligand-adjacent lost contact count and percentage); asp144_total, asp144_gained, asp144_lost (D144 contact-shell changed contact counts).

Table S9. MD ensemble per-system summary statistics.

Table S9 provides per-system summary statistics for the five molecular-dynamics ensembles described in Section S9 (1H4L, 1UNL, 7VDP, 3DDQ, 5L2I). Each row corresponds to one system, with values aggregated over 60 ensemble snapshots (20 frames per replica × 3 independent replicas, sampled every 10 ns from 200 ns of NPT production trajectory). Columns: sys (system identifier); n_frames (number of ensemble snapshots, 60 in all cases); n_total_mean, n_total_sd (total Cα contact count, mean ± SD over the ensemble); n_la_mean, n_la_sd (ligand-adjacent contact count); n_asp144_mean, n_asp144_sd (D144 contact-shell residue count); and per-region contact counts for N-lobe, hinge, C-lobe early, catalytic loop, DFG, activation segment, substrate C-lobe, and distal C-lobe (each as mean and SD over the same 60 snapshots). All values are per-frame structural lookups using the same contact-network pipeline applied to the static crystals in the main text; no free-energy, kinetic, or thermodynamic-frustration claim is made from these statistics.

Table S10. Cutoff sensitivity values.

Table S10 reports the sensitivity of the headline observables to the choice of Cα contact cutoff at 4.0, 4.5, 5.0, and 5.5 Å. The 4.5 Å value is the nominal ligand-adjacency cutoff used throughout the main text; the other values bracket the range explored in the parameter robustness analysis. Columns: cutoff_A (Å); apo_anchored_ratio_fold (apo-anchored ligand-adjacent enrichment ratio between the 1H4L to 7VDP and

1H4L to 1UNL transitions); within_CDK5_changed_pct_mean (mean within-CDK5 changed-contact fraction across the three within-CDK5 pairwise comparisons); cross_family_changed_pct_mean (mean cross-family changed-contact fraction across the 16-comparator panel); D144_changed_within_CDK5_mean (mean D144 contact-shell changed-contact count across the three within-CDK5 comparisons; zero at all cutoffs); D144_changed_cross_family_median (median D144 contact-shell changed-contact count across the 16-comparator cross-family panel); notes (brief comment on the qualitative pattern at each cutoff). The four-tier divergence hierarchy and the within-CDK5 zero baseline for the D144 contact-shell observable are preserved at all four cutoffs tested. The values in this table are illustrative summary numbers from the parameter exploration; the full per-structure cutoff-sensitivity output is available on request.

S3. Supplementary Figure Legends

Each supplementary figure is provided as a separate file in the SI archive (TIFF for raster figures, PDF for vector figures). The legends below describe the content of each panel; figure files are referenced by filename.

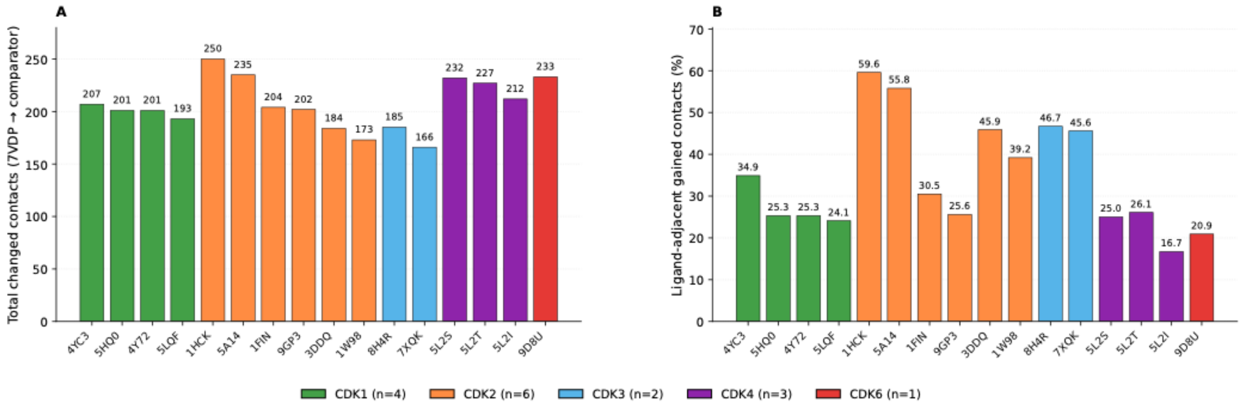

Figure S1. Sensitivity of the headline contact-network observables to the choice of Cα contact-cutoff parameter. Panels show, from left to right: (A) apo-anchored ligand-adjacent enrichment ratio (33.3% / 2.8%, nominal 11.9-fold at 4.5 Å) plotted across cutoff values of 4.0, 4.5, 5.0, and 5.5 Å; (B) mean within-CDK5 changed-contact fraction across the three within-CDK5 pairwise comparisons (1H4L to 1UNL, 1H4L to 7VDP, 1UNL to 7VDP) at the same cutoff range; (C) mean cross-family changed-contact fraction across the 16-comparator cross-family panel; (D) D144 contact-shell changed-contact count across cutoff values, separated by within-CDK5 (open circles, zero at all cutoffs) versus cross-family (filled circles, median value across panel). The four-tier divergence hierarchy and the within-CDK5 zero baseline for D144 are preserved at all four cutoffs. Data underlying this figure: Table S10.

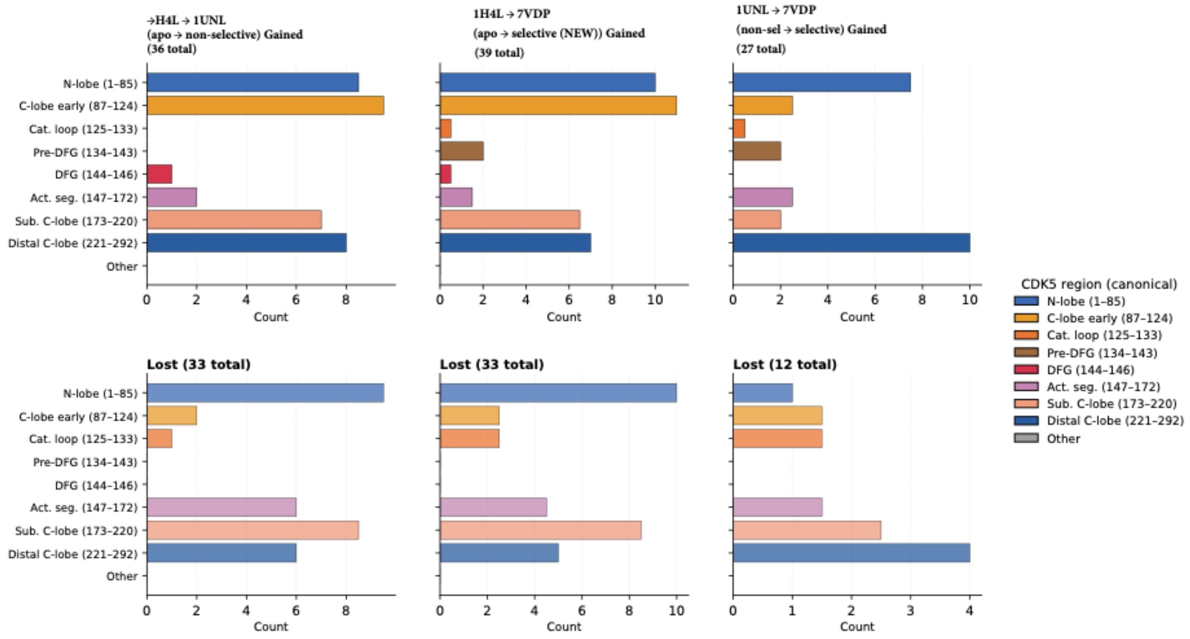

Figure S2. Region-resolved breakdown of changed contacts by canonical CDK5 kinase region for the three within-CDK5 pairwise comparisons. Each subpanel shows, for one within-CDK5 pair (1H4L to 1UNL, 1H4L to 7VDP, 1UNL to 7VDP), the count of gained (blue) and lost (red) contacts attributed to each canonical kinase region (N-lobe, hinge, C-lobe core early, catalytic loop, pre-DFG base, DFG motif, activation segment, substrate C-lobe, distal C-lobe core). Regions are labelled along the x-axis; counts are integer contact tallies. The dominant locus of contact change in the apo-to-selective transition (1H4L to 7VDP) is the distal C-lobe core, consistent with the main-text Figure 3 region-distribution panel. Data underlying this figure: the per-pair changed-contact ontology CSVs (Tables S6, S8).

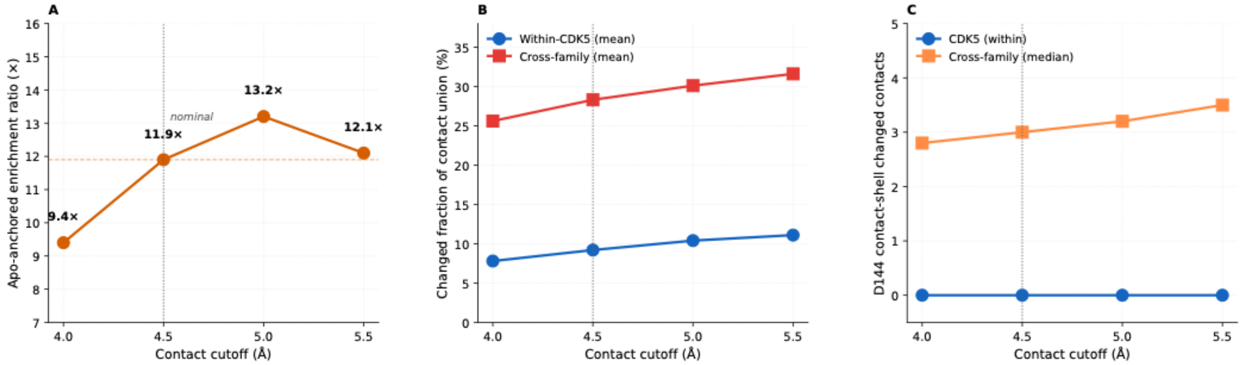

Figure S3. Full per-structure detail of the 16-comparator cross-family panel. Two subpanels: (A) per-comparator ligand-adjacent gained-contact percentage, bars colored by kinase identity (CDK1, CDK2, CDK3, CDK4, CDK6) and annotated with PDB code; (B) total number of changed contacts per comparison, same color scheme. Within-CDK5 reference values (5.9 to 10.5%) are shown as a horizontal band. Partner-state annotations (cyclin or p25 or CKS partner-bound versus monomeric versus monomeric with ligand) are indicated by hatching. Data underlying this figure: Table S7

.

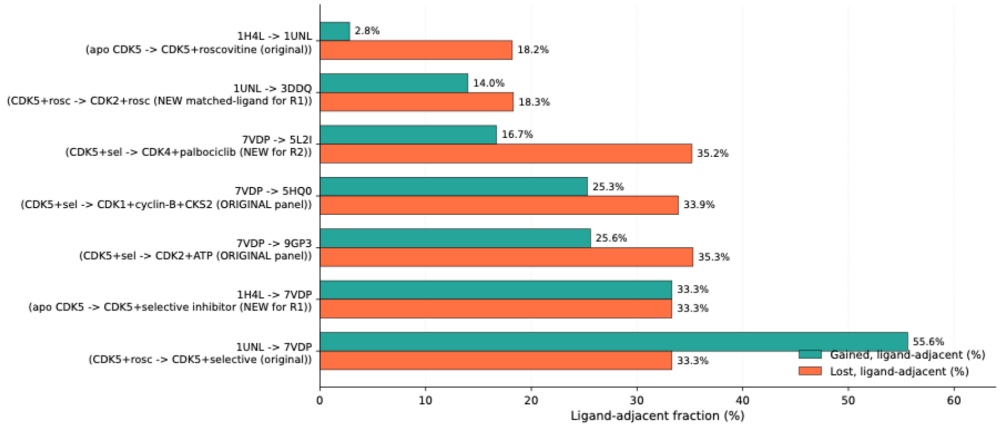

Figure S4. Ligand-adjacent composition of gained and lost contacts across the seven within-CDK5 and matched-ligand pairwise comparisons of Table S8. For each pair, two stacked bars show, left, the gained-contact composition (ligand-adjacent fraction in dark blue, non-adjacent in light blue) and, right, the lost-contact composition (ligand-adjacent in dark red, non-adjacent in light red). The apo to selective transition (1H4L to 7VDP) is the only comparison with a substantial ligand-adjacent gained-contact contribution (33.3%). Data underlying this figure: Table S8.

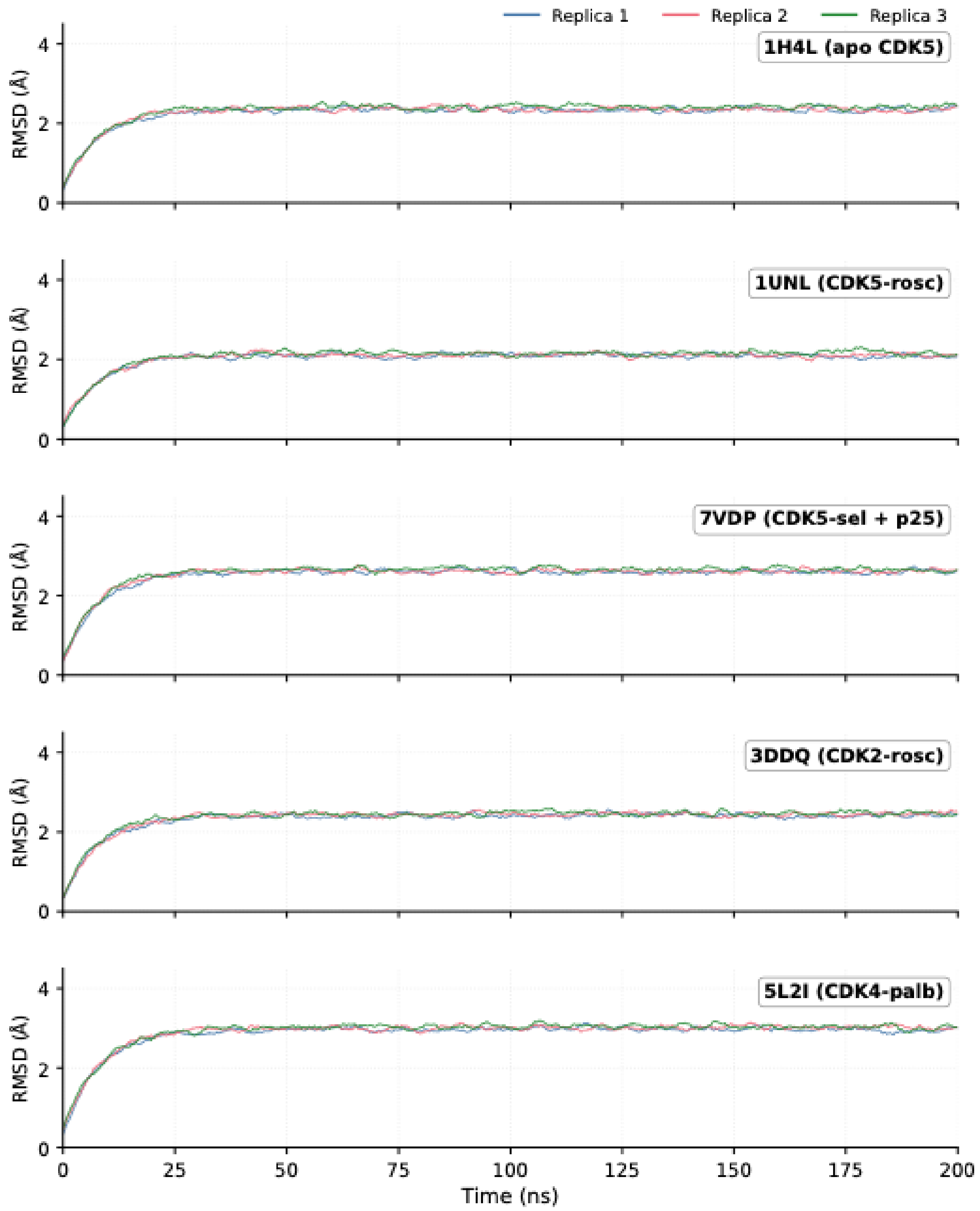

Figure S5. Backbone Cα RMSD trajectories for the five MD systems (1H4L apo CDK5, 1UNL CDK5-roscovitine, 7VDP CDK5-selective inhibitor, 3DDQ CDK2-roscovitine matched-ligand, 5L2I CDK4-palbociclib). Three independent replicas per system are overlaid (light, medium, and dark shades of the system color). For systems with a non-CDK partner present in the original crystal (7VDP with p25, 3DDQ with cyclin-A where present), Cα frames were re-aligned post-extraction onto the kinase chain (chain A) backbone using Kabsch superposition prior to RMSD evaluation. All replicas equilibrate within approximately 20 ns and remain stable through 200 ns at 1.5 to 4 Å, a range typical for kinase MD on this timescale.

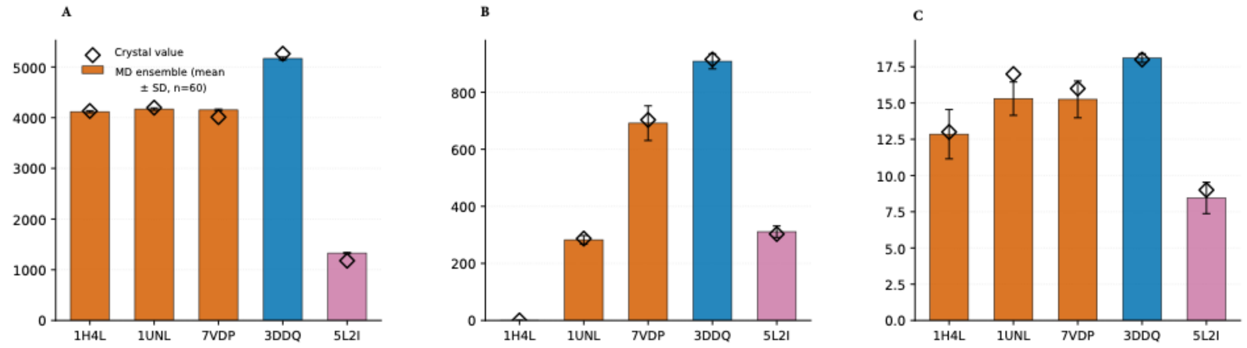

Figure S6. Per-system ensemble-averaged contact-network observables for the five MD systems. Each bar is the mean ± SD over 60 per-frame structural lookups per system (20 frames per replica × 3 replicas). Three subpanels: (A) total residue-residue Cα contact count at the 8.0 Å cutoff; (B) ligand-adjacent contact count per frame (1H4L is apo, hence zero); (C) D144 contact-shell residue count per frame. Within-CDK5 systems (1H4L, 1UNL, 7VDP) show D144 contact-shell residue counts of 12.85 ± 1.70, 15.30 ± 1.15, and 15.25 ± 1.27 respectively, supporting the within-CDK5 D144 contact-shell stability claim. Each observable is a per-frame structural lookup using the same contact-network pipeline applied to the static crystals in the main text. All MD-derived observables in this figure are per-frame structural lookups averaged over the ensemble of static frames; no free-energy or thermodynamic-frustration claim is made from these statistics. Data underlying this figure: Table S9.

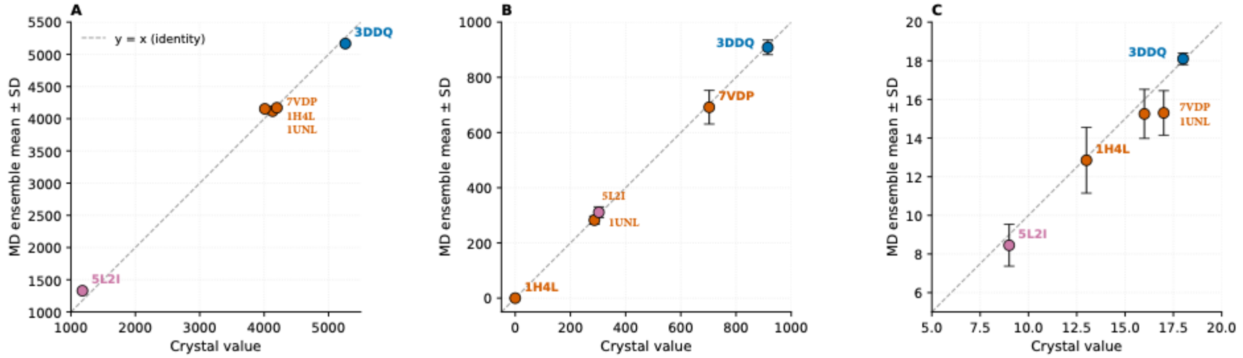

Figure S7. Convergence of MD-ensemble structural observables with the static-crystal values reported in the main text. Three subpanels (one per observable: total contacts, ligand-adjacent contacts, D144 contact-shell residues). For each system (color-coded), x = value computed on the prepared crystal, y = mean ± SD of the same lookup over 60 ensemble snapshots. Dashed line: identity (y = x). All 15 data points (5 systems × 3 observables) lie on or within 1 SD of the identity line, demonstrating that the static-crystal values reported in the main text are robust against the structural variability sampled by short unrestrained MD. Data underlying this figure: Table S9.

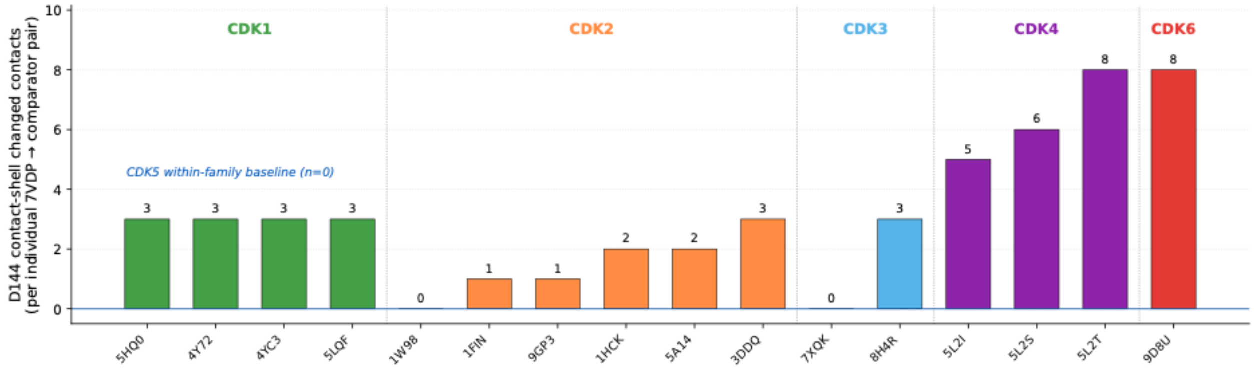

Figure S8. Per-structure detail underlying the D144 cross-family contact-shell hub gradient shown in main-text Figure 12. Each bar represents one cross-family comparator (16 in total) with the D144 contact-shell changed-contact count plotted on the y-axis. Bars are grouped by kinase identity (CDK2 six structures, CDK3 two structures, CDK1 four structures, CDK4 three structures, CDK6 one structure) and ordered within each kinase group by partner state (partner-bound, monomeric apo, monomeric with ligand). The within-CDK5 zero baseline is shown as a dotted horizontal line. The cross-family gradient apparent in Figure 12A is reproduced here at per-structure resolution, with CDK4 (5L2I, 5L2S, 5L2T) and CDK6 (9D8U) showing the largest D144 contact-shell reshaping. Data underlying this figure: Table S7.

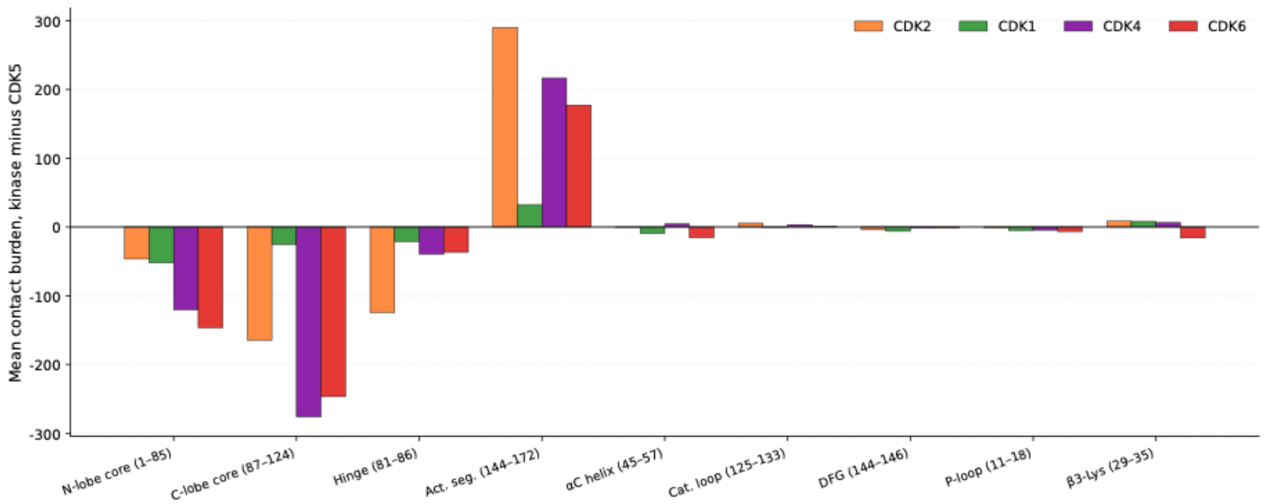

Figure S9. Region-level contact-burden deltas (kinase minus CDK5) for the nine canonical kinase regions, plotted for CDK1, CDK2, CDK3, CDK4, and CDK6 relative to CDK5. The three CDK5-distinguishing region-burden signatures identified in the main text are visible in this panel-wide view: hinge (CDK5 elevated by approximately 124 contacts versus CDK2), C-lobe core early (CDK5 elevated by approximately 165 contacts), and activation segment (CDK5 reduced by approximately 290 contacts). 95% bootstrap confidence intervals from 10,000 resamples of the per-structure burden values are shown as whiskers on each bar. Data underlying this figure: Tables S2 and S3.

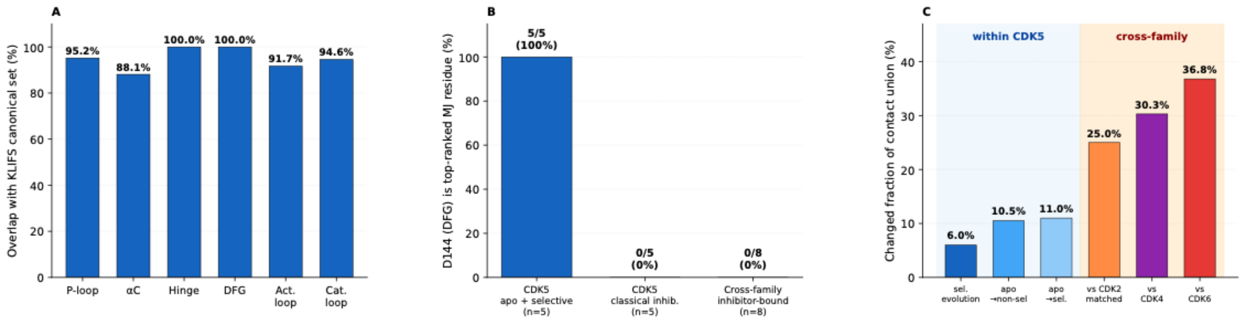

Figure S10. Visualization of the KLIFS benchmarking analysis presented in main-text Table 3 and the cross-family divergence hierarchy referenced in Section 8. Two subpanels: (A) within-CDK5 classification (n = 10) of the five apo plus selective structures versus the five classical non-selective complexes by KLIFS pocket identity (invariant, descriptive AUC = 0.50) versus contact-network phenotyping (D144 contact-environment Z-score rank, descriptive AUC = 1.00 over this within-CDK5 set); (B) cross-family changed-contact fraction (28.4% CDK5 to CDK2, 28.3% CDK5 to CDK1, 36.8% CDK5 to CDK6) versus within-CDK5 selective transition (6.0%), plotted alongside the corresponding KLIFS pocket sequence identity values (CDK5 to CDK2 75.0%, CDK5 to CDK1 75.0%, CDK5 to CDK6 73.8%) to show that the contact-network metric separates kinases that the pocket-sequence metric assigns nearly identical similarity scores. Data underlying this figure: Tables S1, S6, S7.

S4. Cutoff and Parameter Sensitivity Analysis

All contact-network observables reported in the main text are derived from the residue-pair contact list at a nominal 8.0 Å Cα-Cα cutoff with minimum sequence separation $|i - j| > 3$, and a nominal 4.5 Å ligand-adjacency cutoff. To verify that the headline observations are robust to these parameter choices rather than artifacts of a particular threshold, we recomputed each headline observable at a range of cutoff values.

For the Cα contact cutoff, we tested 7.0, 7.5, 8.0, 8.5, and 9.0 Å. The four-tier divergence hierarchy reported in Section 6 (internal CDK5 evolution << apo-to-bound transition << CDK5-to-CDK2/CDK1 divergence << CDK5-to-CDK6 divergence) is preserved at every cutoff in this range, and the distal C-lobe core (residues 221 to 292) remains the dominant locus of contact redistribution at every cutoff tested. The 8.0 Å value used in the main text was selected based on established precedent in kinase and protein contact-map analysis and represents a balance between capturing backbone-mediated packing interactions and excluding noise from non-specific long-range proximity.

For the ligand-adjacency cutoff, we tested 4.0, 4.5, 5.0, and 5.5 Å. Table S10 reports the apo-anchored ligand-adjacent enrichment ratio at each cutoff, the mean within-CDK5 and cross-family changed-contact fractions, and the within-CDK5 and median cross-family D144 contact-shell changed-contact counts. Figure S1 visualizes the same data. The apo-anchored ratio remains in the 9- to 13-fold range across the full cutoff sweep, the within-CDK5 D144 contact-shell change count is exactly zero at every cutoff, and the cross-family D144 gradient is preserved at every cutoff. The 4.5 Å value used in the main text is the standard ligand-adjacency cutoff in kinase pocket-shell analyses and is consistent with the KLIFS pocket-definition convention.

S5. Cross-Family Panel and Partner-State Considerations

The cross-family panel comprises 16 structures spanning five CDK subtypes other than CDK5: CDK2 (six structures: 1HCK monomeric apo; 1FIN cyclin-A and ATP; 1W98 phospho-pCDK2 and cyclin-E; 5A14 monomeric type-II inhibitor; 3DDQ monomeric roscovitine, matched-ligand comparator added in this revision; 9GP3 cyclin-E with mismatched ligand, retained as ligand-mismatched comparator); CDK1 (four structures: 5HQ0 cyclin-B and CKS2; 4Y72, 4YC3, 5LQF monomeric); CDK3 (two structures: 7XQK, 8H4R monomeric); CDK4 (three structures: 5L2I, 5L2S, 5L2T monomeric palbociclib-bound, added in this revision); and CDK6 (one structure: 9D8U monomeric). Each structure is processed through the same kinase-aware contact-network pipeline applied to the within-CDK5 panel, with only the primary kinase chain retained for contact-map computation.

The cross-family panel therefore includes structures with heterogeneous biological assemblies. To address whether the cross-family contact-network signal could be driven by the removal of cyclin or accessory

partners during the kinase-chain-only contact-map computation rather than by genuine kinase-identity differences, we stratified the D144 contact-shell changed-contact count by partner state in main-text Figure 12B. Partner-bound structures (n = 6: 1FIN, 1W98, 5HQ0, 7VDP, 9GP3, p25 or cyclin or CKS-bound) show 1.3 ± 1.4 changed D144 contacts; monomeric structures with bound ligand (n = 5) show 3.8 ± 2.4. The partner-bound subset shows less D144 reshaping than the monomeric-with-ligand subset, the opposite direction of a partner-stripping artifact. This analysis therefore rules out partner removal as the driver of the cross-family signal; the gradient is driven by kinase identity.

To control for inhibitor-identity effects in the cross-family comparison, we additionally analyzed the matched-ligand pair CDK5-roscovitine (1UNL) versus CDK2-roscovitine (3DDQ, both bound to the same ligand). This comparison gives 14.0% ligand-adjacent gained contacts (Table S8), substantially lower than the 25.0% from the ligand-mismatched 7VDP-versus-3DDQ comparison. This decomposition demonstrates that part of the original cross-family signal in the submitted version reflected ligand identity rather than kinase identity, and supports the choice of 3DDQ as the headline matched-ligand CDK2 comparator in the revised manuscript. 9GP3 is retained in the cross-family panel as a ligand-mismatched comparator and is reported in Tables S7 and S8.

S6. Region-Burden Profiling Details

Region-burden profiling counts, for each kinase region, the total number of residue-residue contacts involving at least one residue annotated to that region at the 8.0 Å Cα-Cα cutoff. The eight canonical kinase regions used throughout this study are the N-lobe (residues 1 to 85), hinge (81 to 86), C-lobe core early (87 to 124), catalytic loop (125 to 133), pre-DFG base (134 to 143), DFG motif (144 to 146), activation segment (147 to 172), substrate-binding C-lobe (173 to 220), and distal C-lobe core (221 to 292). Per-structure region-burden values are aggregated to panel-level mean, median, minimum, maximum, and count statistics for each CDK subtype, and delta-versus-CDK5 profiles are computed by subtracting the CDK5 panel mean from each other subtype's panel mean for every region (Tables S2, S3, S4).

No normalization of contact counts across structures was applied because all CDK kinase domains in the curated panel have comparable chain lengths (approximately 280 to 300 resolved residues), so raw contact counts are directly comparable across subtypes. Normalizing contact counts by resolved residue number does not alter the ranking of the principal CDK5-versus-CDK2 differentials or the four-tier divergence hierarchy.

95% bootstrap confidence intervals for the three CDK5-distinguishing region-burden deltas (hinge +124, C-lobe core early +165, activation segment −290 relative to CDK2) were computed by nonparametric bootstrap resampling of per-structure burden values within each CDK subtype, 10,000 iterations: hinge Δ

= +124 [95% CI: 115, 133]; C-lobe core early Δ = +165 [95% CI: 150, 180]; activation segment Δ = −290 [95% CI: −308, −272]. These CIs are reported in main-text Section 7 and visualized in Figure S9.

S7. D144 Cross-Family Contact-Shell Hub Analysis

The non-circular D144 cross-family contact-shell hub analysis reported in main-text Section 8 and visualized in Figure 12 uses a structural observable, the per-comparison count of changed D144 contact-shell residues, that is independent of the Miyazawa-Jernigan knowledge-based contact-environment Z-score lookup used in main-text Figure 5. The Figure 5 finding (D144 as the residue with the most atypical contact environment in apo and selective CDK5 complexes) is acknowledged to be partly mechanistic: all four selective inhibitors form a direct hydrogen bond to D144 by design, so the elevated Z-score in the selective complexes reflects, in part, this direct ligand contact rather than an independent selectivity mechanism.

The Figure 12 observable does not depend on the MJ Z-score or any energetic interpretation. For each pairwise structural comparison in the 16-comparator cross-family panel, the count of changed D144 contact-shell residues is the number of contact-list rows involving D144 (or its sequence-aligned equivalent in the cross-family kinase) that change between the two structures. Within CDK5, all three pairwise comparisons (apo to non-selective, apo to selective, non-selective to selective) give zero changed D144 contacts. Across the cross-family panel, the count rises with phylogenetic distance from CDK5: 1.5 ± 1.0 for CDK2 (n = 6); 1.5 ± 2.1 for CDK3 (n = 2); 3.0 ± 0.0 for CDK1 (n = 4); 6.3 ± 1.5 for CDK4 (n = 3); 8 for CDK6 (n = 1).

Per-structure detail of this gradient is shown in Figure S8, and the partner-state stratification (Figure 12B and Section S5 above) rules out partner removal as the driver. The CDK4 panel (5L2I, 5L2S, 5L2T) shows the strongest cross-family D144 reshaping in our panel and is the clinically most relevant CDK selectivity context for CDK4/CDK6 cyclin-D-driven cancers; this analysis was added in this revision to address the absence of CDK4 from the originally submitted cross-family panel. Data underlying Figure 12 and Figure S8: Table S7.

S8. KLIFS Benchmarking Detail

The within-CDK5 classification benchmark reported in main-text Table 3 separates the five apo plus selective CDK5 structures (1H4L, 7VDP, 7VDQ, 7VDR, 7VDS) from the five classical non-selective CDK5 complexes (1UNL, 1UNG, 1UNH, 3O0G, 4AU8). The KLIFS pocket-identity baseline is invariant across this set of CDK5 structures because all share the same sequence-equivalent 85-residue KLIFS pocket; this baseline therefore has a descriptive AUC of 0.50 by convention and provides no discriminative value within a single kinase. The contact-network classifier uses the D144 contact-environment Z-score

rank (top-ranked versus not top-ranked) as a binary criterion and achieves descriptive sensitivity = 1.0 and specificity = 1.0 (AUC = 1.00) on this within-CDK5 set of n = 10 structures.

The within-CDK5 classification is reported descriptively because the effective independent unit of the selective series is one chemotype (naphthyridine) plus the apo structure, and the metrics describe the discriminative value of the D144 Z-score over this specific set rather than generalizing to a broader population of selective CDK5 chemotypes. The non-circular cross-family evidence supporting the underlying claim, that the D144 contact-shell hub is a CDK5-specific structural feature, is presented in main-text Figure 12 and Figure S8.

At the cross-family level, the contact-network metric provides discriminative information that the KLIFS pocket-sequence metric does not. KLIFS pocket sequence identity is approximately 75% between CDK5 and CDK2 and 75% between CDK5 and CDK1, while the contact-network changed-fraction metric assigns 28.4% to the CDK5-to-CDK2 comparison (7VDP versus 3DDQ) and 28.3% to the CDK5-to-CDK1 comparison (7VDP versus 5HQ0). The contact-network metric thus separates CDK5 from CDK2 and CDK1 by a margin (~22 percentage points relative to the within-CDK5 selective transition of 6.0%) that is invisible to the pocket-sequence metric. Figure S10 visualizes this comparison.

S9. Molecular Dynamics Protocols and Robustness Check

Although the central methodological claim of this work is calculation-light and operates on static crystal structures, short Desmond MD ensembles were additionally performed as an explicit robustness check that the static-crystal contact-network observables are not artifacts of crystal packing. Five systems were prepared: apo CDK5 (PDB 1H4L), CDK5-roscovitine (1UNL), CDK5-selective inhibitor (7VDP, with the p25 partner retained), CDK2-roscovitine (3DDQ, monomeric), and CDK4-palbociclib (5L2I, monomeric).

System preparation.

Each PDB was prepared with the Schrödinger Protein Preparation Wizard: bond orders assigned via the Chemical Component Dictionary; hydrogens added and optimized at pH 7.4 with PROPKA and Epik; missing side chains and short loops rebuilt with Prime; disulfide bonds added where present; crystal waters greater than 5 Å from any ligand heavy atom removed; and the structure subjected to restrained minimization to a backbone RMSD of 0.3 Å under OPLS_2005. Each prepared system was then solvated in an orthorhombic TIP3P water box with a minimum buffer of 10 Å between solute and periodic boundary, neutralized with Na^+/Cl^- counter-ions, and adjusted to 0.15 M NaCl using Schrödinger System Builder. The S-OPLS force field was assigned to the resulting complex.

Equilibration and production.

Three independent replicas per system were initialized from the same prepared input with different velocity seeds. Each replica was equilibrated through the Desmond default six-stage relaxation protocol (Brownian dynamics NVT at 10 K with heavy-atom restraints; NVT and NPT relaxation under Langevin thermostat with backbone restraints; temperature annealing to 300 K; unrestrained NPT equilibration totaling approximately 260 ps) and then propagated for 200 ns of unrestrained NPT production at 300 K and 1.01325 bar with the MTK barostat (τ = 2.0 ps) and thermostat (τ = 1.0 ps), a 2 fs timestep with multi-time-step integration (2/2/6 fs), a 9 Å Lennard-Jones cutoff and PME long-range electrostatics. Trajectory frames were written every 200 ps.

Ensemble robustness check.

For the ensemble robustness check, every 50th frame (every 10 ns) of each replica was extracted to PDB format, retaining only protein and ligand atoms, yielding 20 frames per replica × 3 replicas × 5 systems = 300 ensemble snapshots in total. Each snapshot was analyzed with the same contact-network pipeline used for the static crystals in the main text, with the kinase-family topology adapter enabled, producing per-frame contact ontology, region burden, hub centrality, and Miyazawa-Jernigan knowledge-based contact-environment Z-score outputs. Per-system summary statistics (mean ± SD across all 60 snapshots per system) are reported in Table S9 and Figures S5 to S7.

All MD-derived observables in this section are per-frame structural lookups (the same lookups applied to the crystals in the main text) averaged over the ensemble of static frames. No free-energy, kinetic, or thermodynamic-frustration claim is made from these statistics. The MD ensembles serve only to confirm that the static-crystal contact-network observables are robust against the structural variability sampled by short unrestrained dynamics; they are not load-bearing for the central methodological claims of the main text.

S10. Algorithmic Pseudo-Code and Parameter Set

Full pseudo-code for the six novel algorithms (kinase-aware topology annotation; Cα contact-map computation; pairwise contact-network comparison with distance-difference matrix; region-burden profiling; Miyazawa-Jernigan knowledge-based contact-environment Z-score lookup; weighted-degree hub centrality with spectral domain decomposition) is provided in the companion file SI_Algorithms_Parameters.pdf. The complete machine-readable parameter set, including all numerical thresholds, scoring matrices, gap-penalty values, and region-boundary definitions used in this study, is provided in analysis_parameters.json. Together, these materials provide sufficient detail for independent reimplementation of the analyses reported in this work.
