# Supplementary material for: Contact-Network Phenotyping of the CDK Family Reveals Selective Distal C‑Lobe Contact Redistribution by Modern CDK5 Inhibitors and a Quantitative Selectivity Landscape against CDK2 and CDK1
Source: J Chem Inf Model. 2026 May 29;66(12):7276–95. doi: 10.1021/acs.jcim.6c00886 (PMC13292211; doi:10.1021/acs.jcim.6c00886)
Supplement: Supplementary file 3 [file ci6c00886_si_003.pdf]

Supporting Information: Algorithmic Pseudo-Code, Detailed Workflows, and Machine-Readable Parameters

Contact-Network Phenotyping of the CDK Family Reveals Selective Distal C-Lobe Contact Redistribution by Modern CDK5 Inhibitors and a Quantitative Selectivity Landscape Against CDK2 and CDK1

Manal A. Nael, Laxman M. Alakonda, Khaled M. Elokely*

Journal of Chemical Information and Modeling

This document provides algorithmic pseudo-code and detailed workflow descriptions for all novel computational methods introduced in this study, along with the complete set of analysis parameters in both narrative and machine-readable (JSON) format. These materials are provided to enable independent reimplementation and ensure reproducibility of the reported results, in accordance with JCIM editorial policy on data sharing and reproducibility of scientific results.

Contents

Algorithm 1. Kinase-Aware Topology Annotation
Algorithm 2. Contact-Map Computation and Annotation
Algorithm 3. Pairwise Contact-Network Comparison
Algorithm 4. Region-Burden Profiling
Algorithm 5. Miyazawa-Jernigan Frustration Analysis
Algorithm 6. Hub Centrality Ranking
Workflow. End-to-End Analysis Pipeline
Appendix. Machine-Readable Parameter File (JSON)

Algorithm 1. Kinase-Aware Topology Annotation

This two-stage algorithm assigns every residue in a kinase chain to a canonical structural element. Stage 1 performs a coarse lobe partition based on normalized sequence position. Stage 2 refines the assignment by scanning for conserved motif anchors and re-annotating local windows around each identified anchor.

```
ALGORITHM KinaseTopologyAnnotation(chain)
  INPUT:  chain = ordered list of residues with (resid, resname, position)
  OUTPUT: annotation[residue] = {kinase_segment, kinase_lobe,
                                kinase_motif_region, reference_bin,
                                assign_method, assign_confidence}

  # --- Stage 1: Coarse lobe partition ---
  N = length(chain)
  FOR each residue r in chain:
    frac = index(r) / N                   # normalized position [0, 1]
    IF frac < 0.29:
      annotation[r].kinase_lobe = "n_lobe"
    ELSE IF 0.27 <= frac <= 0.30:
      annotation[r].kinase_lobe = "hinge_region"
    ELSE IF 0.30 < frac < 0.49:
      annotation[r].kinase_lobe = "c_lobe"
    ELSE IF 0.49 <= frac <= 0.59:
      annotation[r].kinase_lobe = "activation_segment"
    ELSE:
      annotation[r].kinase_lobe = "c_lobe"
    annotation[r].assign_method = "coarse_partition"

  # --- Stage 2: Motif-anchor refinement ---
  seq = concatenate(resname for r in chain)

  # 2a. Glycine-rich loop (P-loop)
  gly_pos = scan_motif(seq, "GxGxxG", region="n_lobe")
  IF gly_pos is None:
    gly_pos = scan_closest_match(seq, "G.G..G", region="n_lobe")
  IF gly_pos is not None:
    FOR r in chain[gly_pos - 2 : gly_pos + 6]:  # window of 8
      annotation[r].kinase_segment = "p_loop_candidate"
      annotation[r].kinase_motif_region = "gly_rich_loop"
      annotation[r].assign_method = "motif_anchor"

  # 2b. Beta3-lysine
  beta3_pos = find_conserved_LYS(chain, offset=gly_pos + 8..12)
  IF beta3_pos is not None:
    FOR r in chain[beta3_pos - 3 : beta3_pos + 4]:  # window of 7
      annotation[r].kinase_segment = "beta3_candidate"
      annotation[r].kinase_motif_region = "beta3_lys_region"
      annotation[r].assign_method = "motif_anchor"

  # 2c. AlphaC-helix acidic residue
  alphaC_pos = find_conserved_GLU(chain, offset=beta3_pos + 7..8)
  IF alphaC_pos is not None:
    FOR r in chain[alphaC_pos - 6 : alphaC_pos + 7]:  # window of 13
      annotation[r].kinase_segment = "alphaC_candidate"
      annotation[r].kinase_motif_region = "alphaC_region"
      annotation[r].assign_method = "motif_anchor"

  # 2d. DFG motif
```

```
dfg_pos = scan_motif(seq, "D.G", region="c_lobe")
IF dfg_pos is None:
  dfg_pos = conservative_DFG_fallback(seq, region="c_lobe")
IF dfg_pos is not None:
  FOR r in chain[dfg_pos : dfg_pos + 3]:  # window of 3
    annotation[r].kinase_segment = "dfg_candidate"
    annotation[r].kinase_motif_region = "dfg_region"
    annotation[r].assign_method = "motif_anchor"

# 2e. APE motif
ape_pos = scan_motif(seq, "A.E", region="downstream_of_DFG")
IF ape_pos is None:
  ape_pos = conservative_APE_fallback(seq)
IF ape_pos is not None:
  FOR r in chain[ape_pos : ape_pos + 3]:
    annotation[r].kinase_segment = "ape_candidate"
    annotation[r].kinase_motif_region = "ape_region"
    annotation[r].assign_method = "motif_anchor"

RETURN annotation
```

Algorithm 2. Contact-Map Computation and Annotation

Computes the distance-based Cα contact map for a single kinase chain and annotates each contact with ligand-adjacency and pocket-lining status.

```
ALGORITHM ContactMap(chain, ligand_atoms, pocket_residues)
  INPUT:  chain = list of residues with CA coordinates
          ligand_atoms = list of ligand heavy-atom coordinates (may be empty)
          pocket_residues = set of pocket-lining residue indices
  OUTPUT: contacts = list of (i, j, distance, ligand_adjacent, pocket_lining)
  PARAMS: CUTOFF = 8.0 Angstrom
          MIN_SEQ_SEP = 4
          LIGAND_CUTOFF = 4.5 Angstrom

  contacts = empty list
  N = length(chain)

  FOR i = 0 TO N-1:
    FOR j = i + MIN_SEQ_SEP TO N-1:
      d = euclidean_distance(chain[i].CA, chain[j].CA)
      IF d <= CUTOFF:
        # Ligand-adjacency annotation
        lig_adj_i = ANY(dist(chain[i].heavy_atom, la) <= LIGAND_CUTOFF
                        for la in ligand_atoms)
        lig_adj_j = ANY(dist(chain[j].heavy_atom, la) <= LIGAND_CUTOFF
                        for la in ligand_atoms)
        lig_adj = lig_adj_i OR lig_adj_j

        # Pocket-lining annotation
        pock = (i in pocket_residues) OR (j in pocket_residues)

        contacts.append((i, j, d, lig_adj, pock))

  RETURN contacts
```

Algorithm 3. Pairwise Contact-Network Comparison

Aligns two kinase chains by sequence, computes the distance-difference matrix (DDM), and classifies every residue-pair contact as shared, gained, or lost.

```
ALGORITHM PairwiseContactComparison(chain_1, chain_2)
  INPUT:  chain_1, chain_2 = two kinase chains with CA coordinates
  OUTPUT: shared, gained, lost = lists of contact tuples
          DDM = distance-difference matrix
          metrics = summary statistics dict
  PARAMS: CUTOFF = 8.0 Angstrom; MIN_SEQ_SEP = 4

  # Step 1: Global sequence alignment
  alignment = NeedlemanWunsch(seq(chain_1), seq(chain_2),
                              matrix=BLOSUM62,
                              gap_open=-10, gap_extend=-0.5)
  aligned_positions = [(i,j) for (i,j) in alignment if i != gap AND j != gap]

  # Step 2: Compute distance matrices on aligned positions
  M = length(aligned_positions)
  D1 = matrix(M, M);  D2 = matrix(M, M)
```

```
FOR a = 0 TO M-1:
  FOR b = a + MIN_SEQ_SEP TO M-1:
    (i1, j1) = aligned_positions[a], aligned_positions[b]
    D1[a][b] = euclidean_distance(chain_1[i1].CA, chain_1[j1].CA)
    D2[a][b] = euclidean_distance(chain_2[i1].CA, chain_2[j1].CA)

# Step 3: Distance-difference matrix
DDM = matrix(M, M)
FOR a, b in upper_triangle(M, min_sep=MIN_SEQ_SEP):
  DDM[a][b] = D2[a][b] - D1[a][b]

# Step 4: Contact classification
shared = []; gained = []; lost = []
FOR a, b in upper_triangle(M, min_sep=MIN_SEQ_SEP):
  in_1 = (D1[a][b] <= CUTOFF)
  in_2 = (D2[a][b] <= CUTOFF)
  IF in_1 AND in_2:      shared.append((a, b, D1[a][b], D2[a][b]))
  ELSE IF NOT in_1 AND in_2: gained.append((a, b, D1[a][b], D2[a][b]))
  ELSE IF in_1 AND NOT in_2: lost.append((a, b, D1[a][b], D2[a][b]))

# Step 5: Summary metrics
S = len(shared); G = len(gained); L = len(lost)
C = G + L; U = S + C
metrics = {
  "shared": S, "gained": G, "lost": L,
  "changed": C, "union": U,
  "changed_fraction": C / U,
  "gained_fraction": G / U, "lost_fraction": L / U,
  "mean_abs_DDM": mean(|DDM[a][b]| for all valid a,b),
  "rms_DDM": sqrt(mean(DDM[a][b]^2 for all valid a,b)),
  "max_abs_DDM": max(|DDM[a][b]| for all valid a,b)
}

RETURN shared, gained, lost, DDM, metrics
```

Algorithm 4. Region-Burden Profiling

Computes per-region contact-burden profiles and cross-family delta comparisons.

```
ALGORITHM RegionBurdenProfile(contacts, topology_annotation, panel_structures)
  INPUT:  contacts[s] = contact list for structure s
          topology_annotation[s] = per-residue kinase-region labels
          panel_structures = dict {CDK_subtype: [structure_ids]}
  OUTPUT: burden[subtype][region] = {mean, median, min, max, count}
          delta[subtype][region] = burden[subtype][region].mean
                                 - burden["CDK5"][region].mean

  REGIONS = ["n_lobe_core", "c_lobe_core", "hinge_region",
             "activation_segment_core", "alphaC_region",
             "catalytic_loop", "dfg_region", "gly_rich_loop",
             "beta3_lys_region"]

  # Step 1: Per-structure burden
  FOR each structure s:
    FOR each region R in REGIONS:
      burden_count[s][R] = count of contacts (i,j) in contacts[s]
        WHERE topology_annotation[s][i].kinase_motif_region == R
           OR topology_annotation[s][j].kinase_motif_region == R

  # Step 2: Panel-level aggregation
  FOR each subtype T in panel_structures:
    FOR each region R in REGIONS:
      values = [burden_count[s][R] for s in panel_structures[T]]
      burden[T][R] = {
        mean: mean(values), median: median(values),
        min: min(values), max: max(values), count: len(values)
      }

  # Step 3: Delta vs CDK5
  FOR each subtype T != "CDK5":
    FOR each region R in REGIONS:
      delta[T][R] = burden[T][R].mean - burden["CDK5"][R].mean

  RETURN burden, delta
```

Algorithm 5. Miyazawa-Jernigan Frustration Analysis

Computes per-residue energetic frustration using the Miyazawa-Jernigan statistical contact potential, yielding Z-scores for each residue.

```
ALGORITHM FrustrationAnalysis(chain, contacts)
  INPUT:  chain = list of residues with resname
          contacts = contact list from Algorithm 2
  OUTPUT: Z_score[residue] = frustration Z-score

  # MJ_MATRIX: 20x20 Miyazawa-Jernigan contact energy matrix
  # (values from Miyazawa & Jernigan, J. Mol. Biol. 1996, 256, 623-644)

  # Step 1: Per-residue contact energy
  FOR each residue r in chain:
    neighbors = {j for (i,j,...) in contacts if i == r.index}
```

```
             UNION {i for (i,j,...) in contacts if j == r.index}
  E_native[r] = SUM(MJ_MATRIX[r.resname][chain[n].resname]
                    for n in neighbors)

# Step 2: Background distribution (shuffle-based)
FOR trial = 1 TO 1000:
  shuffled_types = random_permutation(resname for r in chain)
  FOR each residue r:
    E_shuffled[r][trial] = SUM(MJ_MATRIX[shuffled_types[r.index]]
                                             [shuffled_types[n]]
                               for n in neighbors_of(r))

# Step 3: Z-score
FOR each residue r:
  mu = mean(E_shuffled[r])
  sigma = std(E_shuffled[r])
  Z_score[r] = (E_native[r] - mu) / sigma
  # Positive Z = frustrated (native energy worse than expected)
  # Negative Z = minimally frustrated (native energy better)

RETURN Z_score
```

Algorithm 6. Hub Centrality Ranking

```
ALGORITHM HubCentrality(chain, contacts)
  INPUT:  chain, contacts (from Algorithm 2)
  OUTPUT: centrality[residue] = weighted degree centrality score

  FOR each residue r in chain:
    neighbors = contacts involving r
    centrality[r] = SUM(1.0 / distance(r, neighbor)
                        for neighbor in neighbors)

  # Rank residues by centrality (descending)
  ranked = sort(chain, key=centrality, descending=True)

  RETURN centrality, ranked
```

End-to-End Analysis Workflow

The complete analysis pipeline proceeds through five sequential phases. Each phase produces structured CSV output that serves as input to the next.

```
WORKFLOW ContactNetworkPhenotyping(pdb_panel, comparison_pairs)

  PHASE 1: STRUCTURE PROCESSING
    FOR each pdb_id in pdb_panel:
      structure = fetch_from_PDB(pdb_id)
      chain = identify_primary_kinase_chain(structure)
      topology = KinaseTopologyAnnotation(chain)        # Algorithm 1
      contacts = ContactMap(chain, ligands, pockets)    # Algorithm 2
      frustration = FrustrationAnalysis(chain, contacts) # Algorithm 5
      hubs = HubCentrality(chain, contacts)             # Algorithm 6
      EXPORT topology, contacts, frustration, hubs as CSV

  PHASE 2: PAIRWISE COMPARISON
    FOR each (pdb_1, pdb_2) in comparison_pairs:
      result = PairwiseContactComparison(chain_1, chain_2)  # Algorithm 3
      changed_ontology = annotate_changed_contacts(
        result.gained, result.lost,
        topology_1, topology_2,
        ligand_adjacency, pocket_status)
      EXPORT result.metrics, changed_ontology as CSV

  PHASE 3: REGION-BURDEN PROFILING
    burden, delta = RegionBurdenProfile(                  # Algorithm 4
      all_contacts, all_topologies, panel_grouping)
    EXPORT burden, delta as CSV (Tables S1-S6)

  PHASE 4: KINASE-COMPARE SUMMARY
    FOR each comparison:
      tabulate gained/lost contacts by kinase-region
      EXPORT kinase-compare summary as CSV

  PHASE 5: VISUALIZATION
    Generate contact maps, DDM heatmaps, bar charts via Matplotlib
    Generate structural figures via PyMOL
```

Machine-Readable Parameter File

The complete parameter set is provided as a separate JSON file (**analysis_parameters.json**) in the Supporting Information. This file contains all numerical cutoffs, algorithmic settings, motif patterns, scoring matrix specifications, and annotation field definitions required to reproduce the analyses. Key parameters include: Cα contact cutoff (8.0 Å), minimum sequence separation (4), ligand-adjacency cutoff (4.5 Å), Needleman-Wunsch alignment with BLOSUM62 scoring matrix (gap open –10, gap extend –0.5), Miyazawa-Jernigan statistical potential for frustration analysis, and the kinase-topology adapter motif patterns and window sizes.
